# Differences in the intrinsic spatial dynamics of the chromatin contribute to cell differentiation

**DOI:** 10.1093/nar/gkz1102

**Published:** 2019-12-12

**Authors:** She Zhang, Fangyuan Chen, Ivet Bahar

**Affiliations:** 1 Department of Computational and Systems Biology, School of Medicine, University of Pittsburgh, Pittsburgh, PA 15213, USA; 2 School of Medicine, Tsinghua University, Beijing 100084, China

## Abstract

Advances in chromosome conformation capture techniques as well as computational characterization of genomic loci structural dynamics open new opportunities for exploring the mechanistic aspects of genome-scale differences across different cell types. We examined here the dynamic basis of variabilities between different cell types by investigating their chromatin mobility profiles inferred from Hi-C data using an elastic network model representation of the chromatin. Our comparative analysis of sixteen cell lines reveals close similarities between chromosomal dynamics across different cell lines on a global scale, but notable cell-specific variations emerge in the detailed spatial mobilities of genomic loci. Closer examination reveals that the differences in spatial dynamics mainly originate from the difference in the frequencies of their intrinsically accessible modes of motion. Thus, even though the chromosomes of different types of cells have access to similar modes of collective movements, not all modes are deployed by all cells, such that the effective mobilities and cross-correlations of genomic loci are cell-type-specific. Comparison with RNA-seq expression data reveals a strong overlap between highly expressed genes and those distinguished by high mobilities in the present study, in support of the role of the intrinsic spatial dynamics of chromatin as a determinant of cell differentiation.

## INTRODUCTION

Advances in chromosome conformation capture experiments in recent years have opened the way to a new line of research where it is possible to have for the first time a physical understanding of gene-gene couplings at the level of the entire chromatin (1–3). More recently, various studies have shown that changes in the chromatin structure are associated with cell development and differentiation (4–7). However, questions remain regarding the type and extent of conservation and/or differentiation of chromatin structure among different cell lineages and how to quantify these differences. Rao *et al.* (8) found that many loop domains (∼100 kb) are conserved not only in different cells but also across species; Dixon *et al.* (4) noted that, although chromatin domain boundaries tend to be stable during cell differentiation, drastic changes in chromatin interactions are observed both within and between domains; Rudan *et al.* (9) found that the CTCF sites, one of the most important determinants of domain boundaries, evolve under two regimes: some CTCF sites are conserved across species, others are significantly more flexible. A recent single cell study showed that while larger chromatin structures compartments are mostly conserved, the structures of topologically-associating domains (TADs) and loops may vary substantially even within the population of the same type of cells (10). All these observations have shown some levels of conservation as well as variation in the chromatin 3D structure or organization of different cells, suggesting a complex dependency on cell type at the 3D genome level.

We recently introduced a topology-based framework, Gaussian Network Model (GNM), to model and analyze the intrinsic dynamics of the chromatin. GNM is an elastic network model that provides an analytical solution for the spectrum of spatial movements collectively accessible to genomic loci (11). This so-called *chromatin intrinsic dynamics* is uniquely defined by the loci–loci contact topology detected in Hi-C experiments under equilibrium conditions. Proximity ligation-based assays are capable of detecting locus–locus contacts genome-wide and provide a contact map for the 3D chromatin structure. The latter constitutes the major input for constructing a GNM representative of the chromosome architecture and predicting a spectrum of normal modes of motion. The normal modes provide rich information about the equilibrium fluctuations in the positions of genomic loci, their spatial covariance, as well as the chromosomal domains where they are embedded (11,12). Equally important is the relative time scales of these motions are predicted, which permits us to distinguish low-frequency (slow) and high-frequency (fast) modes. Slow modes are usually associated with the cooperative movements of large substructures, and therefore referred to as *global* modes; whereas fast modes correspond to local movements, and hence referred to as *local* modes. Applications to biomolecular structures demonstrated that global modes robustly mediate domain movements relevant to function, whereas local motions confer specificity (13,14).

Cell identity is determined by lineage-specific gene expression during differentiation (15). The process of gene expression is regulated by the accessibility of the corresponding region of the DNA to transcription factors and co-factors. However, numerous studies with biomolecular assemblies have demonstrated that accessibility to binding substrates does not necessarily map to functionality. A more important feature that enables function is the malleability of the putative active sites to optimize binding energetics and support adaptability to structural changes, manifested by conformational flexibility under physiological conditions (16). By analogy, it is reasonable to expect that genes located in loci distinguished by large amplitude fluctuations under equilibrium conditions would be more amenable to processing and expression. We perform here a systematic comparative analysis to examine the existence of such correlations between the 3D mobilities of the genes and their expression levels. Using gene-set enrichment data based on RNA sequencing experiments deposited in Gene Expression Omnibus (GEO) (17,18), we demonstrate the existence of a strong coupling between cell-specific highly mobile genes (HMGs) predicted here by the GNM and the highly expressed genes (HEGs) compiled in the ARCHS4 database (19).

Overall, this present analysis shows that the structural dynamics of chromosomes is an important feature that defines cell identity, in addition to sequence properties; and network models for characterizing 4D genome dynamics provide a computationally efficient platform for assessing cell type-specific behavior at the level of the entire chromatin.

## MATERIALS AND METHODS

### Dataset

The Hi-C datasets used in this study were downloaded from various sources (summarized in Table 1) using the Juicer/Straw tool (20). Preprocessing steps are described in our earlier work (11).

**Table 1.** Dataset of cell lines analyzed in the present study*

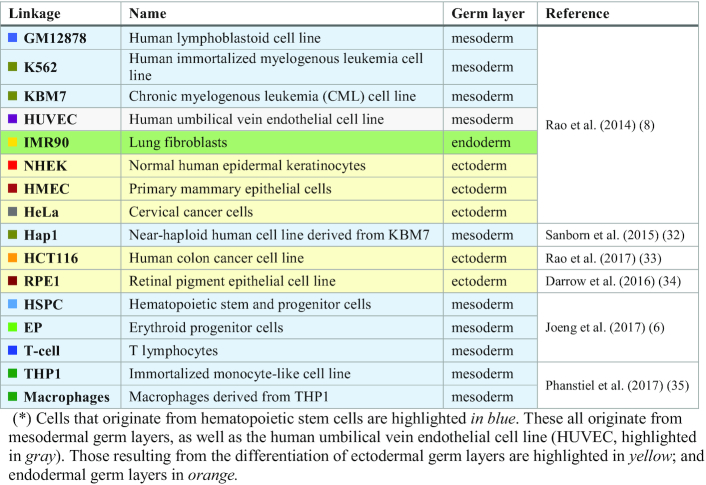

### Gaussian network model

As described earlier (11), the application of the GNM to Hi-C data permits us to construct a network of *n* nodes each representing a gene locus, connected by springs the force constant of which is given by the elements of the Kirchhoff matrix (21,22).

(1) $$\begin{equation*}{\Gamma _{ij}} = \left\{ {\begin{array}{@{}*{2}{l}@{}} { - {M_{ij}}}&{{\rm{if\ }}i \ne j}\\ {\mathop \sum \limits_{j,j \ne i} {M_{ij}}}&{{\rm{if\ }}i = j} \end{array}}. \right.\ \end{equation*}$$

Here ${M_{ij}}{\rm{\ }}$represents the number (or strength) of contacts observed in Hi-C experiment between loci *i* and *j*. The present study uses population-averaged Hi-C data. We note that the GNM is suitable, and even developed, for modeling the equilibrium dynamics of densely packed systems using their ensemble-averaged data. The theory has been shown to be applicable to polymer networks (23,24), to proteins/DNA molecules (13,21,22) including proteins resolved in different forms (25) or ensembles of sequentially heterogeneous proteins that belong to the same family (26), and recently to chromatin (11). The elastic springs simply account for the Gaussian distribution of inter-node distances around their mean values, the nodes being polymer chain ends/cross-links, protein/DNA residues, or genomic loci, in the respective cases. Contact topology is the only ingredient and is compatible with an ensemble of conformers that retain the node positions. The underlying assumption of Gaussian fluctuations for the nodes is mathematically correct as the size of the network increases (using the central limit theorem). The inter-locus contact occurrence/probability obtained from population-averaged Hi-C data, permits to assign stronger effective spring constants to pairs of loci whose interactions are more conserved among the individual cells that compose the ensemble.

Eigenvalue decomposition of ${{\bf \Gamma }}$ yields $n - 1$ nonzero normal modes of collective motions. Each mode *k* is characterized by an eigenvector ${{\boldsymbol{u}}_k}$and an eigenvalue ${\lambda _{k\ }}$ assigned a number as ${\lambda _1} \le {\lambda _2} \le \ldots \le {\lambda _{n - 1}}$. ${{\boldsymbol{u}}_k}$ is an *n*-dimensional normalized vector that represents the collective spatial displacements of all *n* nodes in mode *k* (also called *mode shape*), and ${\lambda _{k\ }}$ scales with the *mode frequency*. The cross-correlation between the displacements ${\rm{\Delta }}{r_i}$ and ${\rm{\Delta }}{r_j}$ of loci *i* and *j* is defined by the *ij*th element of the covariance matrix ${{\bf C}},$

(2) $$\begin{equation*}{C_{ij}} = \langle{\rm{\Delta }}{r_i} \cdot {\rm{\Delta }}{r_j}{\rm{\ }}\rangle = \mathop \sum \limits_{k = 1}^{m - 1} \frac{1}{{{\lambda _k}}}\ {\left[ {{{\boldsymbol{u}}_{k{\rm{\ }}}}{\boldsymbol{u}}_k^{\rm{T}}} \right]_{ij}},\end{equation*}$$

where the angular brackets designate the summation over *m* representative modes. The term $\frac{1}{{{\lambda _k}}}[ {{{\boldsymbol{u}}_{k{\rm{\ }}}}{\boldsymbol{u}}_k^{\rm{T}}} ]$ represents the contribution of mode *k* to **C**. Likewise, the *i*thdiagonal element of ${{\bf C}}$ describes the mean-square fluctuation (MSF) of the *i*th locus around its mean position, i.e.

(3) $$\begin{equation*}\langle{\rm{\Delta }}r_i^2\rangle = {C_{ii}}{\rm{\ }} = \mathop \sum \limits_{k = 1}^{m - 1} \frac{1}{{{\lambda _k}}}\ {\left[ {{{\boldsymbol{u}}_{k{\rm{\ }}}}{\boldsymbol{u}}_k^{\rm{T}}} \right]_{ii}}.\end{equation*}$$

${[ {{{\boldsymbol{u}}_{k{\rm{\ }}}}{\boldsymbol{u}}_k^{\rm{T}}} ]_{ii}}$ plotted as a function of locus index *i* describes the normalized distribution of loci square displacements driven by mode *k* (as $\mathop \sum \limits_i {[ {{{\boldsymbol{u}}_{k{\rm{\ }}}}{\boldsymbol{u}}_k^{\rm{T}}} ]_{ii}}$= *tr*$[ {{{\boldsymbol{u}}_{k{\rm{\ }}}}{\boldsymbol{u}}_k^{\rm{T}}} ]$*=* 1, by definition). The size/amplitude of MSF profile along mode *k* scales with 1/*λ_k_*, lower frequency modes making larger contributions. The term 1/*λ_k_*thus serves as a *statistical weight* for the contribution of mode *k* to $\langle {{\rm{\Delta }}r_i^2} \rangle$ or $\langle {{\rm{\Delta }}{r_i} \cdot {\rm{\Delta }}{r_j}} \rangle$.

In the current study, a uniform resolution of 50 kb per gene locus is adopted for efficient comparative analysis of 16 types of cells. At this resolution, the number of modes accessible to a given chromosome, e.g. in cell line GM12878, varies between 677 (chromosome 22) and 4452 (chromosome 1); and the entire chromatin has access to 56 392 modes. Our previous analysis repeated with different subsets of modes (11) showed that the first $m \approx 100$ softest modes predominantly determine the chromosomal dynamics and yield robust results in good agreement with chromatin accessibility data as well as results derived from higher resolution (5 kb/locus) data. Here we adopted *m* = 500 as a sufficiently large subset to represent the collective dynamics (see Supplementary Figure S1), while also saving computing time, given that the time cost of the Hungarian algorithm used for mode-mode matching (see below) is of the order of $O( {{n^3}} )$.

Directional cross-correlations, or correlation cosines, between the motions of loci *i* and *j* are defined as

(4) $$\begin{equation*}{D_{ij}} = \frac{{{C_{ij}}}}{{\sqrt {{C_{ii}}} \times \sqrt {{C_{jj}}} }}.\end{equation*}$$

Note that ${D_{ij\ }}$varies in the range [−1, 1], the upper and lower limits referring to fully correlated and fully anticorrelated pairs. Anticorrelated pairs undergo correlated/concerted movements along the same direction but opposite senses. ${D_{ij}} = \ 0$ for uncorrelated pairs of loci. In the present study we evaluated ${D_{ij}}$ values for each chromosome.

### Mode-mode overlaps

To compare two sets of modes defined as $\{ ( {\lambda _k^A,{\boldsymbol{v}}_k^A} )\ |\ k \in [ {1,\ {n_A}} ]\}$ and ${\rm{\{ }}( {\lambda _l^B,{\boldsymbol{v}}_l^B} ){\rm{|}}\ l \in [ {1,\ {n_B}} ]\}$ obtained for the same chromosome of two different cell types *A* and *B* for example, we evaluate the *mode-mode overlaps* organized in a correlation cosine map $S( {A,B} )$ the *kl*th element of which is

(5) $$\begin{equation*}\ {\left[ {S\left( {A,{\rm{\ }}B} \right)} \right]_{kl}} = \left| {{\boldsymbol{v}}_k^A \cdot {\boldsymbol{v}}_l^B} \right|\end{equation*}$$

The absolute value is used because the direction of fluctuations (or sense of eigenvectors) is immaterial. ${[ {S( {A,{\rm{\ }}B} )} ]_{kl}}$ varies in the range [0, 1], the lower and upper limits referring to no overlap and complete overlap, respectively.

### Mode conservation across different types of cells

The level of conservation of mode *k* is evaluated by averaging ${[ {S( {A,{\rm{\ }}B} )} ]_{kk}}$ over all pairs (A, B), i.e.

(6) $$\begin{equation*}{\left\langle S \right\rangle _k} = \frac{{N\left( {N - 1} \right)}}{2}\ \mathop \sum \limits_A \mathop \sum \limits_{B,B \ne A} {\left[ {S\left( {A,B} \right)} \right]_{kk}},\end{equation*}$$

where *N* is the total number of cell types (*N* = 16 here).

### Covariance overlap

The similarities between covariance matrices ${{{\bf C}}_A}$ and ${{{\bf C}}_B}$ for respective cell types *A* and *B* is quantified by the *covariance overlap* (27):

(7) $$\begin{eqnarray*}&&L\ \left( {A,\ B} \right) \nonumber\\ &&= \ 1 - \ {\left[ {\frac{{\mathop \sum \nolimits_{i\ = \ 1}^{n - 1} (\sigma _i^A + \sigma _i^B) - 2\mathop \sum \nolimits_{i\ = \ 1}^{n - 1} \mathop \sum \nolimits_{j\ = \ 1}^{n - 1} {{(\sigma _i^A\ \sigma _j^B\ )}^{\frac{1}{2}}}\ {{({\boldsymbol{v}}_i^A \cdot {\boldsymbol{v}}_j^B)}^2}\ \ }}{{\mathop \sum \nolimits_{i\ = \ 1}^{n - 1} (\sigma _i^A + \sigma _i^B)}}} \right]^{\frac{1}{2}}}.\nonumber\\ \end{eqnarray*}$$

Here ${\sigma _{i\ }}$denotes the variance of mode *i*, equal to the reciprocal of ${\lambda _i}$. Because Hi-C maps are measured for different cell lines that may have different total read counts, we normalized the variances as

(8) $$\begin{equation*}\ {w_k} = \frac{{{\sigma _k}}}{{\mathop \sum \nolimits_{l = 1}^{n - 1} {\sigma _l}}}\ = \ \frac{{{{{1}/{\lambda_k }}}}}{{\mathop \sum \nolimits_{l\ = \ 1}^{n - 1} {{{1}/{\lambda_l }}}}}.\end{equation*}$$

${w_k}$serves as a *prior probability* of contribution from mode *k*. This normalization permits us to directly compare the covariance matrices derived from different datasets. Computations were performed using *ProDy*, a Python Application Programming Interface (API) designed for normal mode analysis of biomolecular systems (28,29).

### Identification of mode-mode-matches across different cell lines

Because of cell-type specific variations in the genome structure, the mode spectra also differ. Pairwise comparisons of the mode sets for different cell lines necessitate the identification of the *equivalent* (best matching) modes, which we carried out as a linear assignment problem (30). Accordingly, we first calculate the mode overlaps ${[ {S( {A,{\rm{\ }}B} )} ]_{kl}} \in [ {0,\ 1} ]$ for all eigenvector pairs (*k, l*) of cells A and B using Equation (5), and then evaluate the cost of matching them, or the distance between the two sets, as $1 - {[ {S( {A,{\rm{\ }}B} )} ]_{kl}}$ and finally select the mode pairs that minimizes the total cost/distance using the Hungarian algorithm (30,31).

### Construction of the cell dendrograms based on 4D genome properties

To this aim, we converted the covariance overlap to an arc distance (covariance distance),

(9) $$\begin{equation*}{d_{cov}}\ \left( {A,\ B} \right) = \ {\rm{arccos}}\left( {L\left( {A,B} \right)} \right),\end{equation*}$$

for each pair (*A, B*) of cells, for all chromosomes. Then, we took the maximum distances across all chromosomes for each cell pair to construct a distance graph ${G_D}$ where the vertices represent the cells, and the edges are weighted by the covariance distances between the corresponding vertices. For characterizing the cellular hierarchy among hematopoietic cells, a minimum-spanning tree (MST) was found using Prim's algorithm (32). This way, cell lines at intermediate stages are treated as internal nodes. For all cells, because of the absence of intermediate cell lines, neighbor-joining (NJ) algorithm (33) was adopted, where all cell lines are treated as terminal nodes.

### Overlap between HMGs and HEGs

*Relative mobilities* of genomic loci are calculated by subtracting from the MSF profile$\langle {\ {\rm{\Delta }}{\boldsymbol{r}}{{_i^2}_q}} \rangle$ of locus *i* in cell type *q* the average over all cell types, i.e.

(10) $$\begin{equation*}{\rm{\Delta \ }}{\left\langle {{\rm{\Delta }}{\boldsymbol{r}}_i^2} \right\rangle _q} = {\left\langle {{\rm{\Delta }}{\boldsymbol{r}}_i^2} \right\rangle _q}\ - \frac{{\mathop \sum \nolimits_{q\ = \ 1}^{16} {\langle\rm{\Delta }}{\boldsymbol{r}}{{_i^2}_q}}\rangle}{{16}}.\end{equation*}$$

Loci with the highest ${\rm{\Delta \ }}{\langle {{\rm{\Delta }}{\boldsymbol{r}}_i^2} \rangle _q}$ (top 10%) are considered as highly mobile, and genes within these loci are called ‘highly mobile genes’ (HMGs) for that cell type *q*. The ARCHS4 database (19) from Enrichr (34,35) contains HEGs data for 125 cell types. We used Jaccard index as a metric to evaluate the overlap between HMGs for cell line *q* and the HEGs for cell line *z* from the ARCHS4 database,

(11) $$\begin{equation*}J\ \left( {HM{G_q},\ HE{G_z}} \right) = \frac{{\left| {\ HM{G_q} \cap HE{G_z}\ } \right|}}{{\left| {\ HM{G_q} \cup HE{G_z}\ } \right|}}\ .\end{equation*}$$

We rank-ordered the cell lines in ARCHS4 from highest to lowest overlap based on this metric and found that $\ J( {HM{G_q},HE{G_z}} )$ values were maximized for $q\ = \ z$.

### Application to single-cell Hi-C data

Mouse single-cell and population Hi-C datasets were obtained from the above mentioned single-cell study (10). Population Hi-C datasets were processed in the same way as described in our earlier work (11). As to single-cell datasets, rather than using the highly sparse Hi-C raw data that would lead to disconnected graphs, we used the 3D structural models optimally resolved by Stevens *et al.* for eight single-cell genome (10). In each case, loci-loci contact frequencies were obtained by reverse calculation without any normalization from the 3D model using the simple relationship (10):

(12) $$\begin{equation*}n\ = {\left( {\frac{k}{d}} \right)^3}\ .\end{equation*}$$

Here, $n$ is the contact frequency, $d$ is the Euclidean distance between pairs of loci, and $k$ is a constant. For comparative purposes, the contact maps constructed for the eight different cells were summed element-wise to obtain ‘combined single-cell data’ representative of the cumulative behavior of the cells. GNM analysis was performed for each single-cell, for the combined single-cell, and for population Hi-C contact map.

## RESULTS

### Computations reveal the shared and specific features of the chromosomal dynamics of different types of human cells

**Figure 1.**
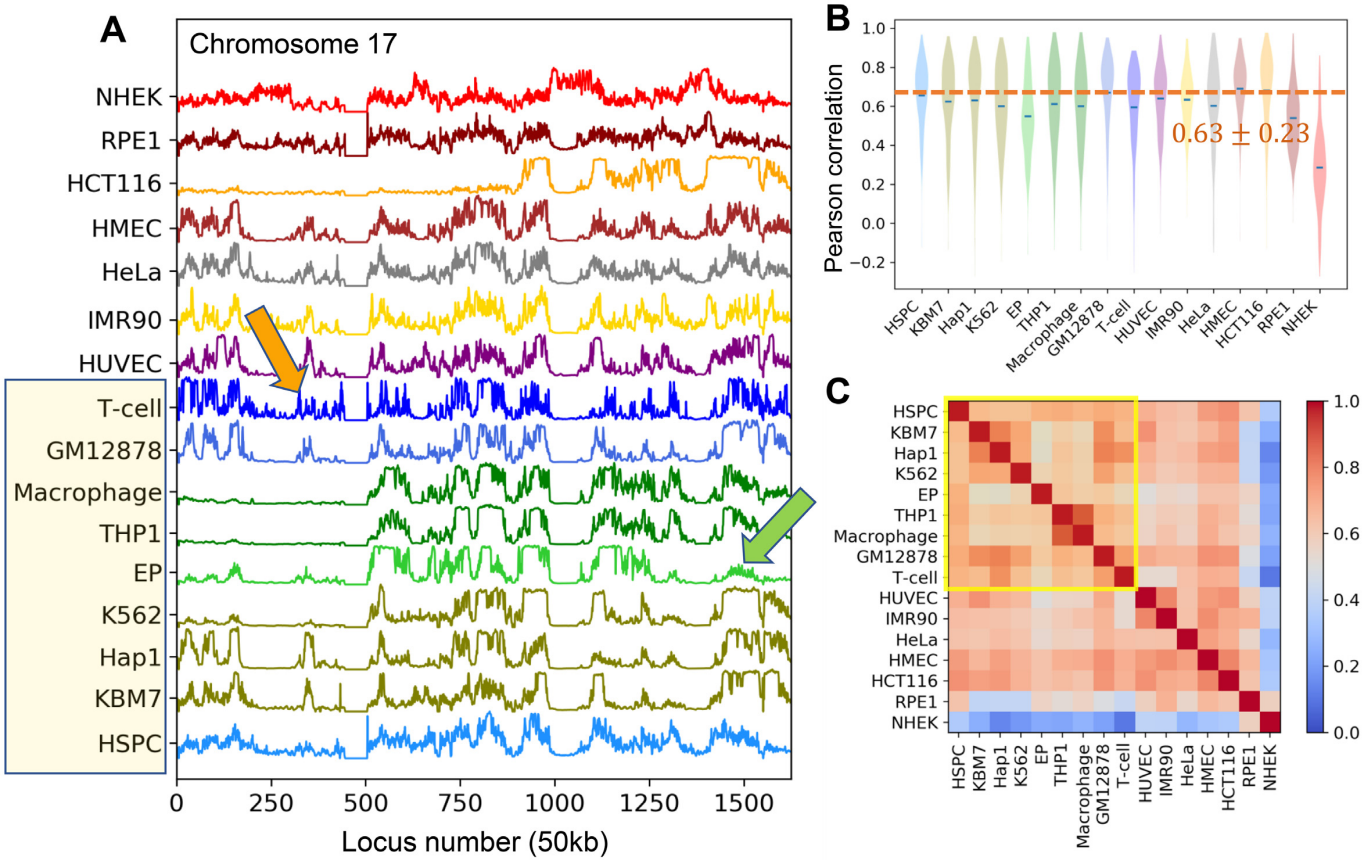
Comparison of the chromosomal dynamics of different types of cells. (**A**) Mobility profile of genomic loci computed for the chromosome 17 of all 16 cells or cell lines in our dataset (Table 1). The curves represent the normalized distributions of mean-square fluctuations (MSFs) in the 3D positions of genomic loci predicted by the GNM, stacked up for visual comparison. (**B**) Violin plots showing the distribution of Pearson correlations between the chromatin mobility profile of individual cells (listed along the abscissa) and all others. *Blue dashes* indicate the mean. (**C**) Similarities between the chromatin dynamics of the examined 16 cell lines. The entries in the heat map show the Pearson correlations between the genomic loci mobility profiles computed for the entire chromatin of each pair of cell lines.

**Figure 2.**
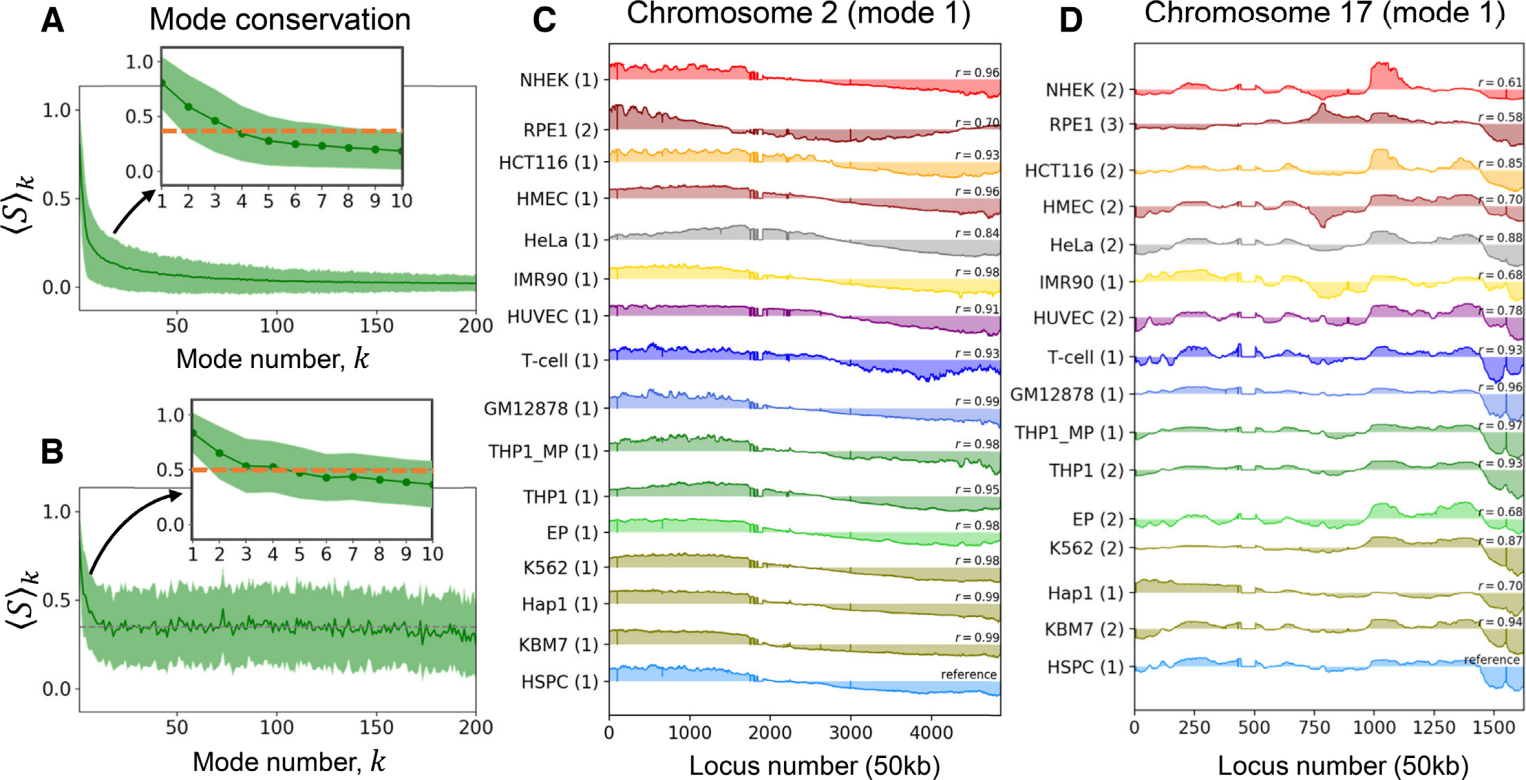
Conservation of the first global mode accessible to the chromatin. (**A**) Mode conservation profile as a function of mode number *k* reveals the high conservation of the global mode ($k\ = \ 1$). The profile displays the correlation cosines between individual mode shapes computed for each of the first 200 modes accessible to the entire chromatin averaged over all cell pairs (Equation 6). The *solid green curve* and the *shade* show the mean and standard deviation, respectively. (**B**) Same as panel A, after reorganizing the modes to select the equivalent modes that best match those of the reference cell, HSPC. Note the higher conservation of modes, but also the accompanying higher variance. See the counterparts of panels A and B for chromosomes 2, 8 and 17 in Figure 3 and Supplementary Figure S3. (C, D) Global mode shape for chromosomes 2 (**C**) and 17 (**D**), highly conserved across 16 cell lines. The curves represent the normalized spatial displacements of loci (abscissa) along the equivalent mode 1 axis. A central hinge region is observed in C at the crossover between positive and negative displacements near loci 2000–2500. The original mode numbers are shown in parentheses on the ordinate, and the correlation cosine with respect to the reference (HSPC) is indicated in each case.

We evaluated the intrinsic chromosomal dynamics of 16 human cells (or cell lines), listed in Table 1, using their inter-loci contact topology data from public Hi-C datasets (6,8,36–39) in the GNM (21,22) framework. We first computed the MSFs of genomic loci for all chromosomes, repeated for all cells. The resulting *mobility profiles* (normalized MSFs, which allow for visual comparison of the behavior of different cells) are illustrated for chromosomes 17 (Figure 1A) and 2 and 8 (Supplementary Figure S2), which show chromosome-specific patterns broadly shared by different types of cells. Note that these are obtained as part of the mobility profiles generated for the entire chromatin, i.e. the underlying GNMs include both intra- and inter-chromosomal contact data for each cell type.

We quantified cell-cell similarities between chromosomal mobility profiles by evaluating pairwise Pearson correlation coefficients. This led to an average Pearson correlation of $r\ = \ 0.63 \pm 0.23$ for cell-cell mobility profiles of all chromosomes (*dashed line* in Figure 1B), except for an ectodermal cell line, NHEK, which correlated poorly with other cells ($r\ = \ 0.29 \pm 0.22$, Figure 1B, *last entry*) but exhibited a correlation with another ectodermal cell line, RPE1 ($r\ = \ 0.58 \pm 0.14$). RPE1, in turn, exhibited relatively strong correlations with two other ectodermal cell lines, HMEC ($r\ = \ 0.66 \pm 0.08$) and HCT116 ($r\ = \ 0.63 \pm 0.12$), as well as the only endodermal cell line, IMR90 ($r\ = \ 0.68 \pm 0.11$).

### The mobility profiles of genomic loci exhibit global similarities while retaining cell-specific patterns

Pearson correlations between the mobility profiles of genomic loci in different types of cells can be viewed in Figure 1C for all pairs of cells. The results refer to the collective fluctuations of all chromosomes (entire chromatin) for each pair of cell types. The hematopoietic cell lines (the *first nine* in Figure 1B, C) exhibit a correlation of at least 0.5 with each other, with three being the most dissimilar, EP (erythroid progenitors), THP1 and THP1-derived macrophages. EP is the only red blood cell line in the dataset, and THP1 and the macrophages are more differentiated than other hematopoietic cell lines. HSPC, the hematopoietic stem and progenitor cells, correlate well with almost all other cell types ($r\ = \ 0.66 \pm 0.17$, Figure 1B, *first entry*) and will be used as reference for quantitative analyses of cell type-specific behavior.

The mobility profiles in Figure 1A and Supplementary Figure S2 show that each chromosome exhibits a unique mobility profile, closely maintained across different cells. A closer look reveals certain localized cell-specific features. For example, despite the similarities between the hematopoietic cell lines (highlighted in *yellow boxes* in Figure 1A and C), some notable distinctions are observed at selected loci: GM12878 and T-cells, both of which belong to lymphoid lineage, and KBM7 and its derivative Hap1, show enhanced fluctuations in the short arm of chromosome 17 (loci 320–380) as compared to other hematopoietic cell lines (see the *orange arrow*); and erythroid progenitor (EP) has more suppressed mobility at the tail of chromosome 17 (loci 1400–1600) than other hematopoietic cells (*green arrow*). Ectodermal cell lines NHEK, RPE1 and HCT116, except for HMEC, exhibit the most distinctive fluctuation patterns compared to cells derived from other germ layers.

### Dissection of mode spectrum reveals the high conservation of global modes

The mobility profiles are obtained from the linear (weighted) combination of square displacements of loci in 3D, contributed each by a representative set of normal modes (1 ≤ *k* ≤ 500). Of interest is to dissect the mode spectrum and analyze the contribution of individual modes to assess the origin of the similarities and dissimilarities between the chromosomal mobility profiles of different cell types. As explained in the Materials and Methods, each mode *k* has a *shape* (*n*-dimensional vector ${{\boldsymbol{u}}_k}$ representing the normalized displacements of the *n* loci along the *k*th mode axis) and a *statistical weight*$1/{\lambda _k}$ which defines the amplitude of the (square) displacement along mode *k*. Modes are assigned *mode numbers* increasing from 1 to $n - 1$ with decreasing amplitude, such that the first few modes (global modes) make relatively large contributions to the fluctuation spectrum; whereas higher modes usually exhibit more localized fluctuations.

**Figure 3.**
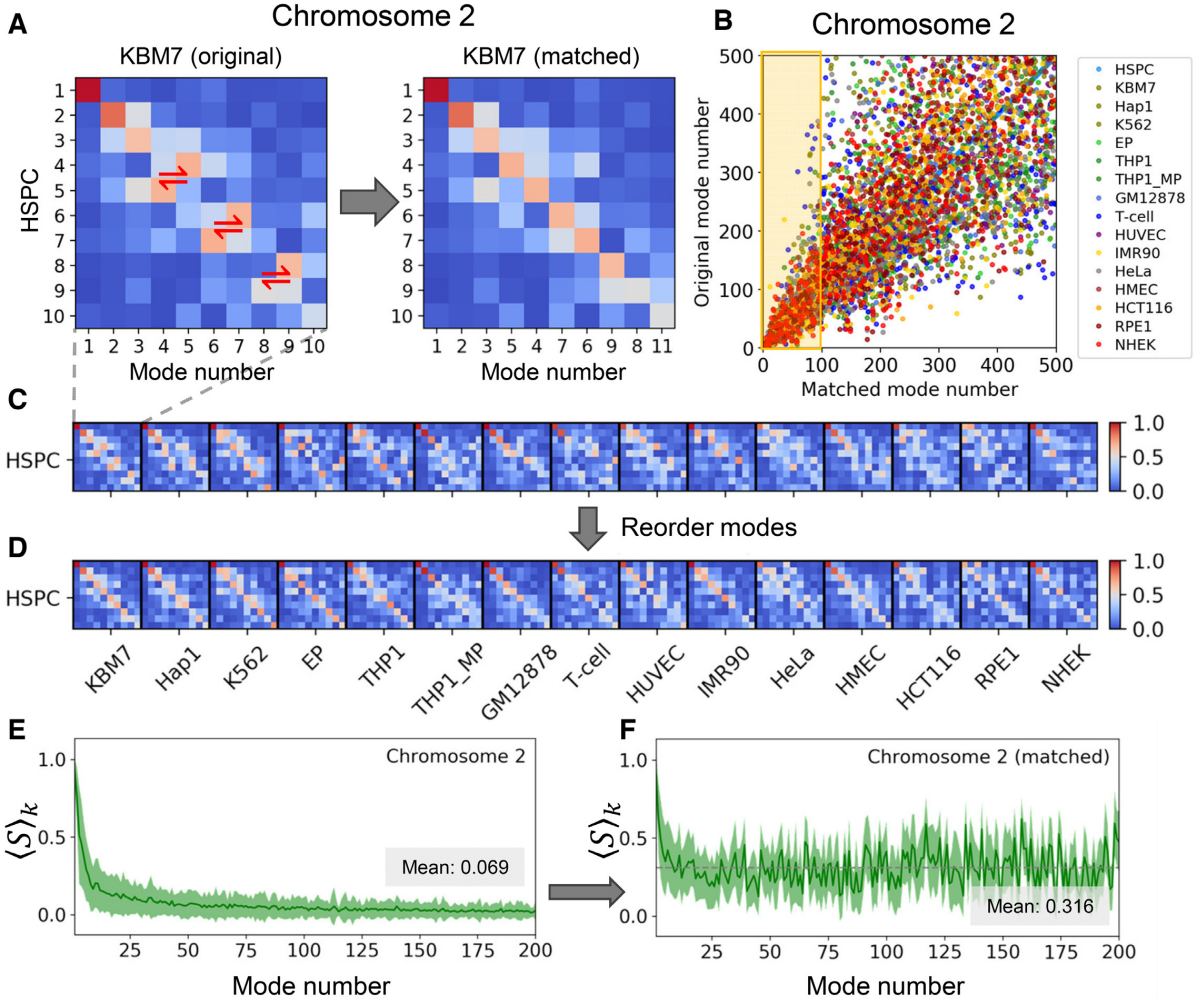
Conservation profile of soft modes and mode-mode overlaps across different cell lines, illustrated for chromosome 2. (**A**) Mode-mode matching process illustrated for the softest 10 modes of HSPC and KBM7. The entries in the heat map are the mode-mode correlation cosines, with the strength of correlations decreasing from *red* to *blue*. *Red two-way arrows* display the modes that are swapped to result in the map *on the right*. (**B**) Comparison of the original (*abscissa*) and reassigned (*ordinate*) mode numbers after optimal matching to the mode numbers of the reference cell (HPSC). Results for different cell types are color-coded, consistent with previous figures. (**C**, **D**) Same as panel (A), displayed mode-mode overlaps between all (15) pairs of cell types and the reference HPSC, and their reordering to identify equivalent modes. (E, F) Conservation level of the first 200 modes before (**E**) and after (**F**) mode-mode matches, similar to Figure 2A, B, but shown here for chromosome 2. The *gray dashed lines* in E and F indicate the respective mean values of 0.069 and 0.316.

Decomposition of the chromosomal mode spectrum accessible to each cell type yielded the *mode conservation* curve presented in Figure 2A as a function of mode number. The ordinate ${\langle S \rangle _k}$ designates the correlation cosine between the shape of mode *k*, averaged over all pairs of cells. The first mode is highly conserved across all examined cells (${\langle S \rangle _1} = \ 0.84 \pm 0.18$) indicating the prevalence of a *global mode shape* for the chromatin, shared by all cell lines; Figure 2C and D illustrate the global mode shape (parts) corresponding to the respective chromosomes 2 and 17. A closer look at mode conservation within individual chromosomes (illustrated for chromosome 2 in Figure 3E, and 8 and 17 in Supplementary Figure S3A, B) also exhibited the same pattern, mainly high-to-moderate conservation of the first few modes (see also the inset, Figure 2A), followed by a rapid decrease with increasing mode number.

We further evaluated the mode-mode overlaps among the first 10 modes, for every pair of cells. The heatmap on the *left* in Figure 3A shows an example of such overlaps for HSPC and KBM7, where each element represents the correlation cosine ${[ {S( {A,{\rm{\ }}B} )} ]_{kl}} = | {{\boldsymbol{v}}_k^A \cdot {\boldsymbol{v}}_l^B} |$ between the *k*th and *l*th modes ($1 \le k,\ l \le 10$) of the respective cells *A* and *B* (in this case HSPC and KBM7). The same type of overlap map is displayed for HSPC and each of the other cell types in Figure 3C and the complete map for all pairs of cells is presented in Supplementary Figure S3C. These maps confirmed that the slowest modes from different cells exhibit relatively high overlaps (see *red pixels* near the upper left part of the diagonal in each block). Even in the case of the most distinctive cell types such as GM12878 and NHEK, the overlap between the top three modes remained above 0.65.

### While different cell types have access to conserved genome-scale dynamics, the ‘active’ modes of motions differ from cell to cell

Closer examination of the heat maps Figure 3A (*left*) and Supplementary Figure S3C (*inset*) reveal that a high mode-mode overlap between different cell lines is not necessarily observed at the diagonal elements of each block, indicating a mismatch in mode numbers between different cells. As mentioned above, the *mode number*/*index* is a physically meaningful quantity, smaller indices referring to lower frequency or larger amplitude modes. Thus, an off-diagonal *red pixel* in the heat map means that the two modes are similar in shape (relative distribution of loci movements during this mode), but not in size (absolute amplitude of motions). In a sense, the mode will be more pronounced or active in one type of cell compared to the other. Here ‘more active’ means a ‘predisposition to undergo a relatively larger displacement’ along that mode (exhibited by the cell with the smaller mode number).

The differences in the mobility profiles of chromosomal loci in different cell types (Figure 1A) can thus originate not only from the different shapes of the modes - evidenced in the comparison of the global ($k\ = \ 1$) mode shapes of chromosomes 2 and 17 for the 16 cells/cell lines in Figure 2C and D), but also from their different statistical weights.

**Figure 4.**
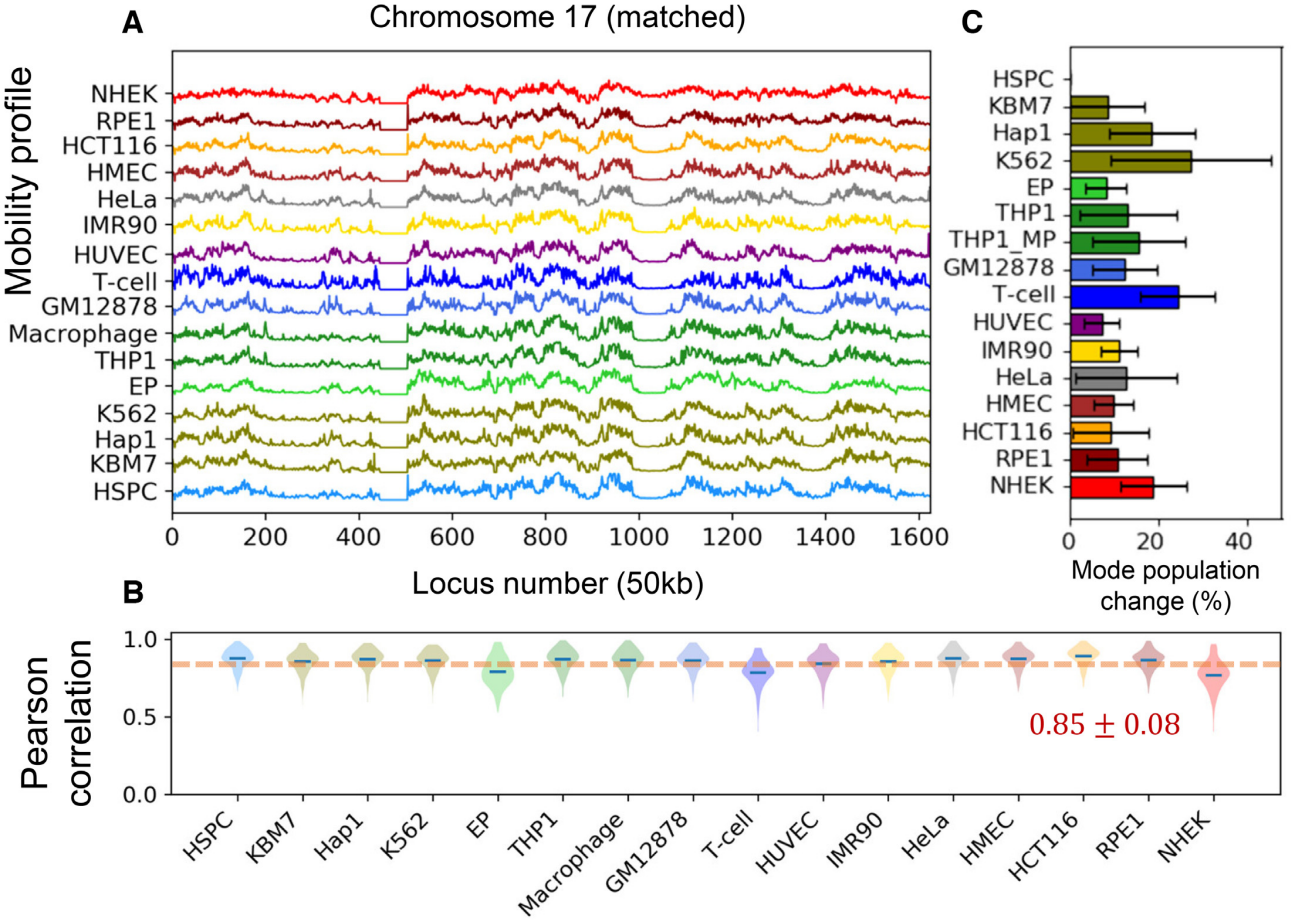
Verification of the close similarity of the spectrum of motions after eliminating the differences originating from the frequency dispersion. (**A**) Mobility profiles of genomic loci on chromosome 17 based on first 500 *matched* modes identified for all cell lines. (**B**) Distribution of Pearson correlations between the chromatin mobility profiles of each cell type and all others, obtained with the same set of modes. (**C**) Percent change in mode population after inclusion of equivalent modes for each cell line, averaged over all chromosomes. The error bars indicate the standard deviation among chromosomes. HSPC has no change because it is used as the reference.

To understand to what extent the frequency dispersion or the selective activation of pre-existing shared modes underlies the differences in the observed spatial mobilities of genomic loci, we adopted the mode numbers of HSPCs as reference (as the most undifferentiated cell in the dataset, based on the mode shape overlaps) and reordered the modes of the other 15 cell lines to achieve the highest mode-mode overlaps. Figure 3A provides a schematic description of mode number reassignment method. Essentially, the shape and frequencies of the modes are retained, but their mode index is changed to match the so-called ‘equivalent’ modes in evaluating the average mode-mode overlaps. This led to heat maps with highest mode-mode overlaps along the diagonals of the blocks, as illustrated in Figure 3D and Supplementary Figure S3F inset, and a conservation profile presented in Figure 2B for the entire chromatin, and Figures 3C and S3D-E for the respective chromosomes 2, 8 and 17. By selectively including the ‘equivalent modes’ (or *matched* modes) and excluding others to evaluate the intrinsic dynamics, we end up with mobility profiles that are almost identical across all cell lines (Figure 4A). The recomputed mobility profiles led to significant increase in the Pearson correlations among different cell lines ($r\ = \ 0.85 \pm 0.08$, Figure 4B; compared $0.63 \pm 0.23$in Figure 1A, B).

It is important to note that the equivalent modes were identified by searching a broader range of modes, and often found from amongst ‘higher’ modes (Figure 3B), which means some of the matched modes had relatively low weights/amplitudes and thus might not be contributing to collective dynamics in a given cell type as effectively as they do in another cell type. Slow modes tended to be retained without significant change in mode numbers; whereas fast modes exhibited large differences. For example, the first 10 matched modes are selected from among the original 20 modes of the cell lines; whereas the top 100 modes of the reference cell line (HSPC) are matched by up to 400 (original) modes of the other cell lines (Figure 3B).

The degree of *collectivity* of a given mode provides a measure of its distribution over different parts of the structure (40). Slower modes are usually more collective, cooperatively involving large groups of loci, and collectivity usually decreases with mode number, but this is not necessarily a smooth decrease. The collectivity of the top 500 modes for all chromosomes and cell types evaluated before and after matching the modes showed that the dependency of collectivity on mode number remained unaffected by mode-mode matching (Supplementary Figure S4).

Overall, these results show that the chromatins of different types of cells have access to comparable modes of motion (encoded by their similar contact topologies) but not all of these pre-existing intrinsic modes are manifested, resulting in cell-specific mobilities of genomic loci. The differences in mobility profiles observed in Figure 1 originate from the fact that the ‘active’ modes differ between different cell lines. If we select the original softest 500 modes, we end up with cell-type specific mobility profiles. The profiles become similar only if we select the ‘equivalent’ modes even though this set contains contributions from relatively ‘inactive’ modes and excludes some ‘active modes’.

In other words, *conserved* modes among different types of cells manifest themselves in a *signature profile* shared among all cell lines (Figure 4A). But in practice, not all modes are operative, and the fluctuations of genomic loci exhibit cell type-specific features. Some modes are ‘mute’ while others are fully deployed, and which modes are selectively deployed depends on the cell type.

It is of interest to assess the fraction of the original modes that have been replaced by equivalent modes. Results are presented in Figure 4C. We note that despite showing the greatest enhancement to conform to the signature profile, NHEK is only in the third place to show largest mode changes (19%), indicating that some of the original slow modes for NHEK greatly differed from those for HSPC, and the substitution of those modes effectively restituted the mobility profile to closely resemble the signature profile. Two cell lines that showed the largest mode changes are K562 (27%) and T-cells (24%). Their mobility profiles exhibited an increase in average correlation with all others from 0.60 (each) to 0.86 and 0.78, respectively. EP experienced the least mode number changes (8.1 ± 4.6%), yet its average correlation increased significantly (from 0.54 to 0.79).

### Locus-locus correlations in 4D show stronger dependency on cell type than do loci mobilities

Locus mobility is a one-dimensional (1D) property representing the size of motions intrinsically accessible to each gene locus; whereas locus-locus spatial coupling/correlation is an additional and maybe more important feature contributing to the chromatin dynamics in a three-dimensional (3D) space. Such interactions between loci can be quantified by the covariance matrix ${{\bf C}}$ (see Materials and Methods). ${{\bf C}}$ is an $n \times n$ symmetric matrix, the *ij*th element of which describes how much the pair of loci *i* and *j* are correlated with regard to their spatial movements, averaged over all possible modes of motions. Such correlations may originate from connectivity (sequence neighbors along the DNA), spatial proximity in the 3D genome or from ‘allosteric’ effects involving other common connections. Upon normalization of ${{\bf C}}$ with respect to the MSFs of the loci, we obtain the directional cross-correlation matrix, ${{\bf D}}$, which describes the correlation cosines, exclusively, between the motions of loci pairs (see Materials and Methods). The mobility profile and directional cross-correlations thus provide complementary information on the respective sizes and orientational couplings of genomic loci movements.

**Figure 5.**
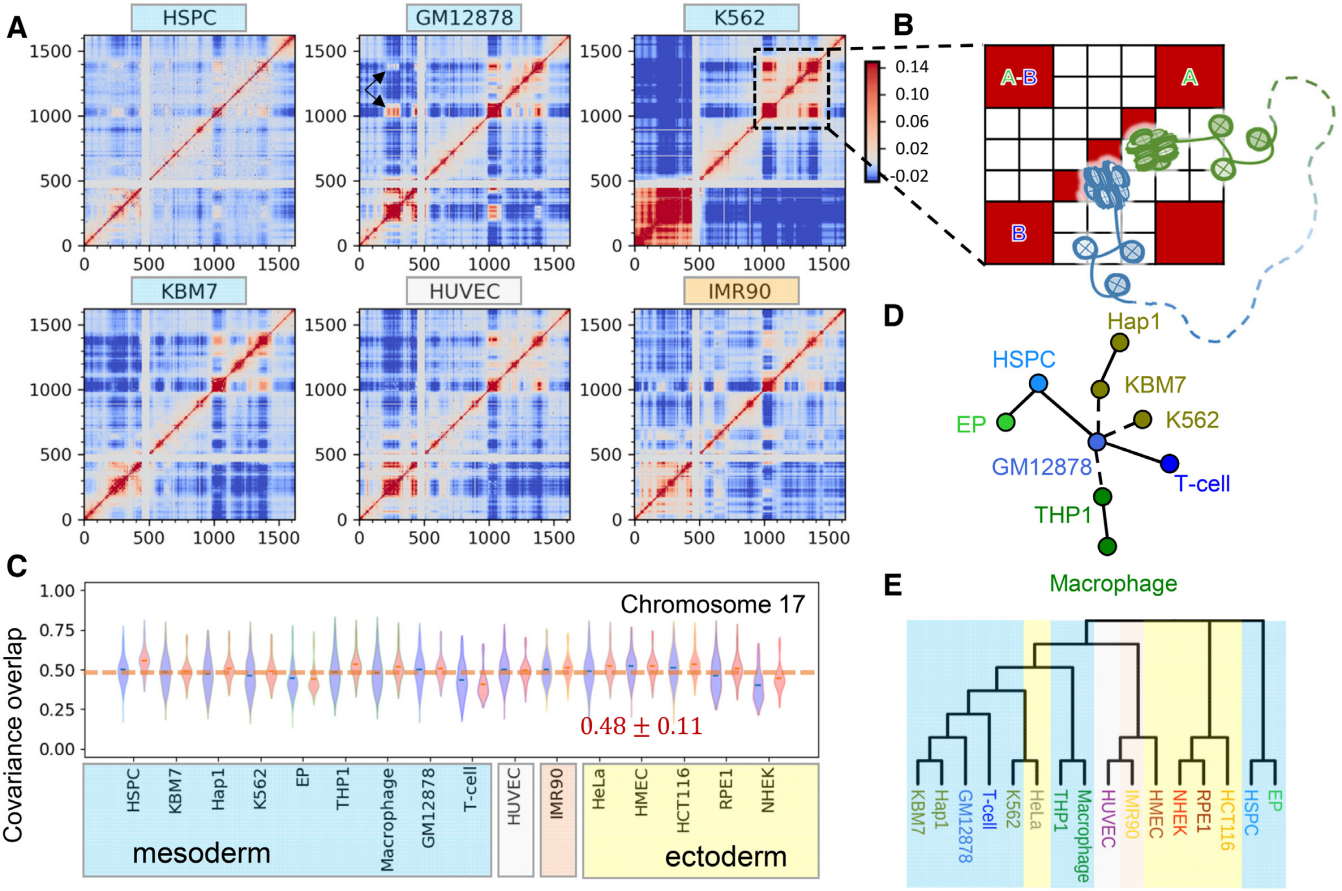
Locus-locus cross-correlations reflect cell-type specificity. (**A**) Directional cross-correlations *D_ij_* between genomic loci computed for chromosome 17, shown for six different cell types (see Supplementary Figure S5 for the remaining ten). *D_ij_* values vary from −0.26 (anticorrelated, off-diagonal regions in *dark blue*) to 1.0 (fully correlated, diagonal elements, in *red*) and color-coded as indicated in the bar to ease the visualization. The *white bands* at loci ∼400–500 refer to the centromere, where Hi-C contact data are missing. (**B**) Schematic description of chromosomal organization indicated by the correlation patterns. *Red blocks* A and B on the diagonal represent two domains (A: *green*; and B: *blue*) with tightly packed DNA; and the off-diagonal *red blocks* indicate the long-range domain-domain couplings between A and B. The *dashed curve* depicts a long sequence not shown between A and B. (**C**) Covariance overlaps among cell lines averaged over all chromosomes based on the first 500 original (*blue violins*) or matched (*red violins*) modes. (**D**) Collective MST for hematopoietic cells based on the covariance overlaps computed for *all* chromosomes (see Equation 10). (**E**) Neighbor-joining tree for all cell lines constructed using the minimum (maximum) covariance overlaps (distances) for all chromosomes. Edge/branch lengths in panels D/E not proportional to arc distances. The color shades are added to facilitate the visualization of the grouping of ectodermal, mesodermal and other cell lines as in C.

Figure 5A and Supplementary Figure S5A show such directional cross-correlation maps for chromosome 17 as an example, computed for all cell lines in our dataset. We observe strong correlations among sequential neighbors (*red band* along the diagonal). Pronounced couplings are observed within selected regions presumably representing TADs (*red squares* on the diagonal). As to the cross-correlations between sequentially distant loci, a range of behavior is detected. For instance, an interesting pattern near the end of the long arm (loci 1000–1500 approximately) is distinguished in K562: two distal domains exhibit a pronounced coupling (highlighted by the *black square* in Figure 5A and schematically depicted in Figure 5B). This behavior tends to be more prominent in mesodermal cell lines (including hematopoietic cell lines labelled in *blue* and HUVEC labelled in *gray*), than in ectodermal cell lines such as NHEK, HMEC, HCT116 and RPE1. We also notice that, as compared to other cell lines, K562, HCT116, THP1 and macrophage exhibit stronger cross-correlations among the loci in the short arm (loci 1–500), implying a higher packing density in the region. Another manifestation of tight packing is the suppressed mobility of loci occupying such regions, noted earlier in the short arm region (Figure 1A). Thus, tightly packed regions which exhibit minimal movements are also distinguished by their close directional couplings, in accord with their restricted movements as almost rigid blocks. This feature can be observed clearly by displaying the MSF profiles along the axes of the cross-correlation maps. The *black arrows* in Supplementary Figure S6 highlight such regions.

We then examined the overlaps between covariance matrices obtained for different cell lines. Unlike fluctuation profiles, covariance matrices showed higher variations among the cells. The overall covariance overlap averaged over all chromosomes and pairs of cell lines was 0.48 ± 0.11 (*blue violins* in Figure 5C). NHEK again yielded the lowest average overlap of $0.40 \pm 0.10$, however, it was not an outlier, and many other cells exhibited comparable values. The overlaps between the covariance matrices could be slightly improved upon mode matching (*red violins* in Figure 5C), but the improvement was much more limited compared to that observed in locus mobility profiles. Overall this analysis the directional couplings between loci movements exhibit a stronger dependency on cell type than that the mobility profiles of individual loci.

### Covariance overlap between loci serves as a discriminative metric for assessing the divergence of cell lines

To understand the impact of cell-type-specific locus-locus dynamical couplings on the response or adaptation of cells to endogenous or environmental effects, on cell differentiation, we quantified the differences between the covariances obtained for individual chromosomes in different cell lines using as metric the covariance overlap (see Materials and Methods) and performed a series of experiments *in silico*.

First, we examined the loop domain loss for HCT116 under the influence of auxin using time-dependent Hi-C dataset, mainly Hi-C maps for HCT116 cells under normal conditions (auxin-), 6 hours after the treatment of auxin (auxin+), and 20, 40, 60 or 180 min of auxin withdrawal (37). We evaluated the covariance overlaps between the covariance (of all chromosomes) of the treated cells at each time point and those of the normal cells. As expected, the average covariance overlap dropped by approximately 30% after the treatment and gradually recovered with time after auxin withdrawal (Supplementary Figures S5B and S7).

Second, we asked whether the differences in covariances could be used to distinguish cell types. To answer this question, we constructed a distance graph for the cell lines based on the covariance overlaps, where each node represents a cell line and each edge is weighted by the arc distance ${d_{cov}}$ between the covariance matrices, obtained for the corresponding pair of cells (see Materials and Methods). We then determined the MST that revealed the relations between cell lines based on their covariance. We applied this procedure to the hematopoietic cell lines because among the cell lines we collected those had the clearest differentiation hierarchy and lineages in earlier or intermediate stages, such as HSPC, GM12878 and THP1. Covariance overlaps obtained for different chromosomes gave rise to different MSTs (Supplementary Figure S8A-C), possibly due to different rates in the spatial organization of different chromosomes during cell differentiation. To determine the MST for all chromosomes, we constructed a graph based on the maximum ${d_{cov}}( {A,\ B} )$ (see Equation 9) between all cell types, which led to the tree presented in Figure 5D. The latter broadly agrees with single-cell transcriptional behavior of hematopoietic progenitors (41,42). For visual comparison, we display a single-cell RNA map for hematopoietic progenitors from earlier work (41) and compare with our MST in Supplementary Figure S9. The tree correctly reproduces the transcriptional similarities among blood cell lineages (indicated by *solid edges*), including the fact that Hap1 is derived from KBM7. K562 and KBM7, both of which relate to myeloid progenitors, should have been closer to HSPC than GM12878, a lymphoblastoid cell line. This discrepancy might originate from the cancerous nature of K562 and KBM7 (hence the *dashed edges* between KBM7, K562 and GM12878). Moreover, the relationship among monocyte progenitors (THP1) and GM12878 is ambiguous, also marked by a *dashed edge* in Figure 5D.

Third, we applied the neighbor-joining method to construct a ‘phylogenetic’ tree based on the maximum covariance distance map (Supplementary Figure S8D) obtained for all investigated cell lines. The resulting tree (Figure 5E) groups together similar cell lines, e.g. hematopoietic cells cluster together except for HSPC and EP, and epithelial cell lines, NHEK, RPE1 and HCT116, are under the same branch. Interestingly, one of the two leukemia cell lines, K562, is clustered with HeLa derived from the cervical tumor, whereas the other, KBM7 and its derivative Hap1, are grouped with normal lymphoid cells, GM12878 and T-cells, suggesting cancer heterogeneity among leukemia cells.

The similarities between cell lines found here at the chromatin dynamics level are in accordance with an earlier study (43) where they found that HMEC, despite being an epithelial cell line originated from the ectoderm, was more similar to endodermal and mesodermal cells; the leukemia cell lines, KBM7 and K562, resemble GM12878; and there are similarities between IMR90 and HUVEC. However, the results for epidermal keratinocytes, NHEK, are different. High similarities between the TADs identified for NHEK and those for GM12878 and K562 were reported (43), while NHEK shows little resemblance to other cell lines in terms of its intrinsic dynamics.

Overall, these results suggest that the pairwise covariance overlaps could be used as a metric for quantifying the differentiation of cells with regard to their collective dynamics. Clearly the present dendrograms obtained by using covariance distances in MST and NJ methods refer to a classification based on global dynamics, and do not imply a developmental or evolutionary relation. However, it would be of interest to examine to what extent the differential expression patterns in different cell types relate to the differences in the intrinsic dynamics (or spatial mobilities) of the genes in those cells, which will be examined next.

### Genes distinguished by high mobility correlate with those highly expressed in a cell-type-specific manner

**Figure 6.**
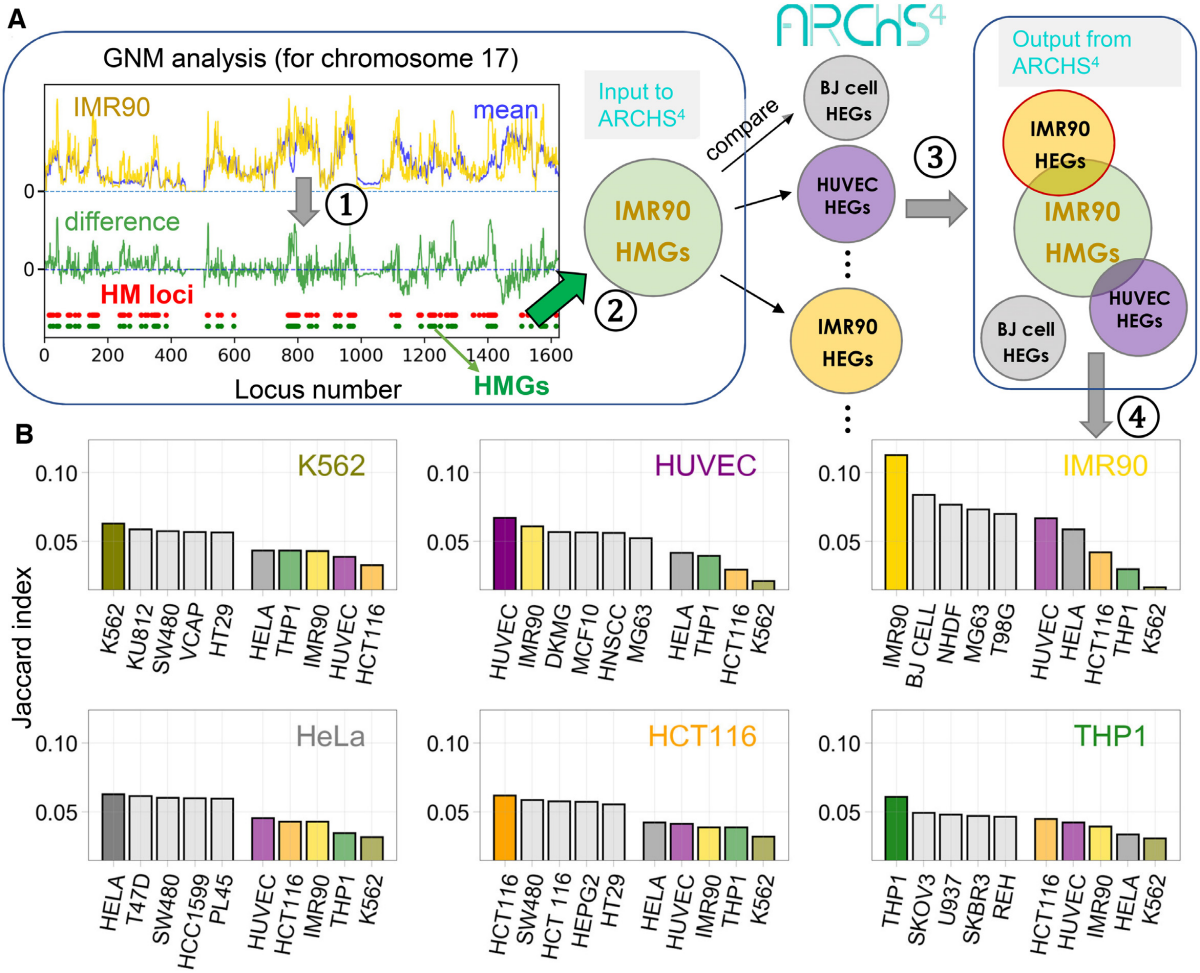
Strong correlation between cell-type-specific highly mobile genes (HMGs) and highly expressed genes (HEGs). (**A**) Illustration of the *4-step* protocol *in silico* test of the relationship between HMGs and HEGs for IMR90 as a query cell: (1) Identification of high mobility (HM) loci from the difference (*green curve*) between the mobility profile obtained with the GNM (shown here for IMR90 chromosome 17; *yellow curve*), and that averaged across all 16 cell lines (*blue curve*); HM loci are defined as those exhibiting the top 10% mobility, shown by the *red dots*. (2) Genes located in HM loci, HMGs, are identified (shown by *green dots*). The procedure is repeated for all chromosomes, and the resulting list of HMGs for IMR90 chromatin is used for screening against the cell-type specific HEGs compiled in the ARCSH^4^ database; (3) Similarities between the HMGs of the query cell type and the HEGs of the 125 cell types in ARCSH^4^ are measured by the Jaccard index, and rank-ordered for each query cell type; (4) Top-ranking five (screened) cell lines whose HEGs exhibit the highest overlap with the HMGs of the query cell are shown by the bar plots in panel B. Results are presented in (**B**) for six of our dataset cell types that were represented in ARCSH4, and the corresponding Jaccard scores are additionally displayed (*right colored bars*) in each case for comparison. The top-ranking cell type (from the pool of 125 in ARCSH4) turns out to be the query cell type itself, demonstrating the distinctive overlap between HMGs and HEGs specific to each cell type.

Chromatin accessibility plays an essential role in regulating gene expression and cell differentiation by allowing or preventing physical interactions between transcription factors or other regulatory proteins and genomic loci (44). Theoretically, the accessibility of a site is predominantly determined by the local packing density, which is manifested by high mobilities in the 3D fluctuation profiles predicted by the GNM (11). A further question is to understand the role of high mobility at selected loci in defining cell differentiation. To this aim, we identified the subset of *highly mobile genes* (HMGs) distinguished by large amplitude motions (peaks in the fluctuations profiles, e.g. Figure 1A) in a given cell type but not in others, and explored the biological relevance of these strong departures from the average fluctuation profile of all cells (Figure 6A), if any, to the differential function of the specific cells.

Toward answering this fundamental question, we compared the HMGs predicted here with the *highly expressed genes* (HEGs) in multiple cell lines annotated in the ARCHS^4^ database (19) (see Materials and Methods). ARCHS^4^ contains information on the HEGs of 125 common cell lines, obtained by integrating gene expression data from RNA-seq experiments deposited in the Gene Expression Omnibus (GEO) (19). These 125 cell lines constitute our pool of ‘*candidate cell types*’, which will be searched to explore the relationship between HMGs and HEGs. The pool contains HEG data for six of the 16 cell lines investigated here (K562, IMR90, HCT116, HUVEC, HeLa, and THP1), which will be referred to as the *query* cell lines. The following computational test/protocol of four steps, schematically described in Figure 6A is adopted: (i) we compute for a given cell type *q* the *relative mobilities* of genomic loci with respect to the average over all cells examined by the GNM, (ii) we identify those loci, or corresponding genes, which rank in the top 10% in terms of their mobility. These are the HEGs specific to cell type *q*. (iii) The list of HEGs is provided as input to search the pool of candidate cell types and extract those cell types *p* whose HMGs provide maximal overlap (highest Jaccard indices) with the HEGs of cell type *q*. (iv) the top-ranking 5–6 candidate cells resulting from this screening process are shown in the bar plots in panel **B** (*left bars*), along with the results for the other GNM-characterized cell types also contained in the pool (color-coded; *right bars*).

The results are presented in Figure 6B for each of the six query cell lines. In each case, we screened the query cell line against the entire dataset of 125 candidate cell lines in ARCHS4 and computed the Jaccard index as a measure of the overlap between the HMGs of the query cell line and the HEGs of the candidate cell lines; and the bars display the top-ranking candidate cell lines whose HEG pattern shows the highest similarity to the HMG pattern of the query cell line. In each case we also display the results for the other five query cell lines for comparative purposes. Notably, the top-ranking candidate cell line turned out to be the query cell line itself in all cases (Figure 6B).

Other top-ranking candidates also bear resemblances to the corresponding target as well. For instance, BJ cell (normal human foreskin fibroblast), NHDF (normal human dermal fibroblast) and MG63 (osteosarcoma with fibroblastic shape) all share a fibroblast-like morphology as IMR90, a fetal lung fibroblast. In the case of THP1, a typical cell model for primary monocytes, one of its top candidates, U937, also shows monocytic traits (Figure 6B).

This analysis clearly demonstrates that (i) the unique HMG pattern predicted here to typify each cell line strongly correlates with the cell-line-specific HEG behavior, suggesting a strong link between high mobility and high expression and (ii) the set of HMGs provides a sufficiently distinctive feature to accurately discriminate between cell lines exhibiting different expression patterns. It also suggests that (iii) high conformational flexibility or spatial mobility may be a prerequisite for enabling productive interaction with proteins and thereby effective transcription or gene expression.

## DISCUSSION

The present comparative study of the intrinsic dynamics of chromosomes in a series of cell lines using corresponding Hi-C data in the Gaussian Network Model (GNM) shed light to several fundamental features, including the shared fluctuation patterns in the spatial positions of different types of cells or *signature dynamics*, evident in the modes of motions in the lowest frequency (and highest collectivity) regime. Even more interesting was the identification of cell type-specific variations in the equilibrium dynamics, originating from the effective contribution of a different pool of modes of motions (in the low-to-intermediate frequency regime) by different types of cells. Thus, even though many mode shapes in this regime bore close similarities between different cell types, their frequency were different; and this resulted in cell-type specific distributions of highly mobile genes (HMGs) in different cell types. A distinctive overlap between HMGs and highly expressed genes (HEGs) has been found upon systematic screening of gene expression data. This relation, with important implications in cell differentiation, invites attention to the significance of the intrinsic mobilities of the individual genes in enabling their transcriptional regulation, shown here using a physical model for the first time at the genome-scale, for several types of cell lines.

Overall, the slowest (and most collective) modes of motion intrinsically accessible to the individual chromosomes of different types of cells were distinguished by their conservation, even among phenotypically divergent cells such as GM12878 and NHEK, yielding an average correlation cosine of ${\langle S \rangle _1} = \ 0.84 \pm 0.18$ between all cell type pairs. The modes in the intermediate and high-frequency ranges, on the other hand, appeared to be less conserved. This is physically reasonable, because global modes, especially the first few, usually underlie the structural stability (13). The spatial organization of chromatin is hierarchical, and recent studies have been focusing on identifying chromosomal domains at different scales as well as the hierarchy itself (45–47). The conservation of global modes may suggest that the cells maintain similar upper levels of the hierarchy but organize the lower levels differently. This type of organization may ensure a stable genome structure *and* the framework to achieve cell type-specific gene transcription/regulation activities.

Careful analysis showed that the differences in the mode spectrum essentially resided in the contributions (statistical weights) of different modes of motions, rather than the availability of these modes of motions. In other words, different types of cells share pre-existing modes with similar ‘shapes’ but different frequencies. While the ensemble of modes intrinsically accessible were comparable, some modes were ‘silent’ in selected cell types, while others were ‘active’. This distinction is reminiscent of the existence of the same set of genes in all cell lines but their differential expression levels in different cell types depending on the specific functions of these cells. Similarly, we have the same ensemble of collective motions theoretically accessible, but not all of them are operative within the same time window, and as a result the different types of cells end up exhibiting differential dynamics (Figure 1A and Supplementary Figure S2). We demonstrated that the 16 cell types presently analyzed would have exhibited the same fluctuation behavior (Figure 4A, B), if their equivalent modes were equally active. However, the differences in their actual amplitudes (quantified by the discrepancy between original and matched mode numbers, shown in Figure 3B and 4C) led to cell-type specific gene mobilities, which directly related to cell-type specific gene expression properties.

Locus mobility is a 1D property and does not reflect the complexity of chromosomal dynamics. So, using it as a measure of cell type specificity would be incomplete because of the absence of inter-loci interactions. Here we compared the covariance matrices using the covariance overlap, which is a well-established metric to compare the subspaces spanned by normal modes and has been used in many applications (27,48,49). We found that while the locus mobilities shared much resemblance among cell lines, long-range couplings measured by spatial cross-correlations (covariance) exhibited more diversities, even among closely related cells (Figure 5A and Supplementary Figure S5A). For example, for chromosome 17, the off-diagonal cross-correlations are much weaker and sparser for K562 than for other hematopoietic cells (Figure 5A and Supplementary Figure S5A), which may suggest that in K562 the two arms of chromosome 17 are partially disordered, if not unfolded. Moreover, in the same chromosome, there are two anchor regions (Figure 5A, *black arrows*) that connect the two arms of the chromosome in GM12878; whereas the regions are absent in two leukemia cell lines, K562 and KBM7, as well as in THP1 and all four epithelial cell lines. These observations agrees with the view that while chromatin domain positions are stable during differentiation, interactions within and between domains can change drastically (4). Furthermore, the cell trees based on covariance overlaps did capture some lineage relationships.

The recently developed database ARCSH4 was used here as a test bed for exploring the relationship if any between cell-type specific HMGs (most mobile genes) and HEGs. For each query cell line, we quantified the overlap between cell-line-specific HMGs and the HEGs collected in ARCHS4, and rank-ordered the best matches using the Jaccard index as a metric. This analysis clearly revealed the strong relationship between the genes distinguished by their high mobility (HMGs) in a given cell type, and those experimentally observed to be highly expressed (HEGs) in that particular cell type, confirmed for six different cell types that were represented in ARCSH4.

Overall, our analysis demonstrates that the cells intrinsically maintain similar mode shapes but reorganize the order (frequency) of modes to achieve different overall mobility, potentially exposing different chromosomal regions for transcription factors and co-factors to access. Such selective operation of different modes of motion amongst a shared pool of pre-existing modes may emerge as a mechanism contributing to cell specificity, reminiscent of the selective expression of functional genes in different cells even though all cells share the same DNA.

The current methodology is computationally efficient and scalable. While the focus here has been the chromosomal dynamics of different types of cells, the GNM lends itself to the analysis of the entire chromatin for different types of cells. Examination of the mode spectrum and mode collectivities for the entire chromatin, illustrated in Supplementary Figure S10 for GM12878, shows that most of the soft modes (>85% by weight, among the first 100) involve intra-chromosomal movements only. Examination of mode shapes corresponding to the highly collective modes (peaks in panel **B**) shows inter-chromosomal coupled movements, as illustrated for modes 10 and 16 are panel **C**.

An exploratory analysis to assess the significance of using as input single-cell Hi-C data, as opposed to those collected for a population of cells, reveals the heterogeneity of the individual cells with regard to their spatial fluctuation patterns. This type of heterogeneities are consistent with the variations in the structures of TADs and loops from cell to cell, as noted earlier (10). Yet, the soft modes of motions predicted based on population-averaged Hi-C data could be detected in single cells, which could be attributed to the conserved/shared organization of larger (A and B) compartments on a genome-wide basis in every cell (10). Supplementary Figure S11 illustrates this feature. In panel A, we compare the soft mode spectrum for single mouse embryonic stem cells chromosome 15 (based on single-cell Hi-C data (10)) with that obtained for a population of the same cell type, which reveals that several soft modes (along the diagonal) are shared. Furthermore, the mode-mode correlations between the soft modes predictions based on single-cell Hi-C data and those based on population-averaged Hi-C data are improved upon grouping together the results for single cells (termed ‘combined single-cell’ in panels B and C). The MSFs of gene loci calculated for the population and for the combined single-cell using the first 100 softest modes also exhibit some level of consistency between two systems (panel D). As more data will become available, more detailed analytical treatments using broader datasets, including more extensive single cell Hi-C data, will help obtain more complete and accurate information on cell-specific chromatin dynamics as well as their relevance to cell differentiation.

## Supplementary Material

gkz1102_Supplemental_File
