# Supplementary material for: Differences in the intrinsic spatial dynamics of the chromatin contribute to cell differentiation
Source: Nucleic Acids Res. 2019 Dec 12;48(3):1131–45. doi: 10.1093/nar/gkz1102 (PMC7026660; doi:10.1093/nar/gkz1102)
Supplement: gkz1102_Supplemental_File [file gkz1102_supplemental_file.pdf]

Supplementary Information for

Differences in the intrinsic spatial dynamics of the chromatin contribute to cell differentiation

She Zhang[1], Fangyuan Chen[1,2] and Ivet Bahar[1,*]

[1]Department of Computational and Systems Biology, School of Medicine, University of Pittsburgh, Pittsburgh, PA, 15213, USA; [2]School of Medicine, Tsinghua University, Beijing, 100084, China

*To whom correspondence should be addressed.
Tel: 1 412 648 3332 Fax: 1 412 648 3163 - bahar@pitt.edu

Supplementary Figures

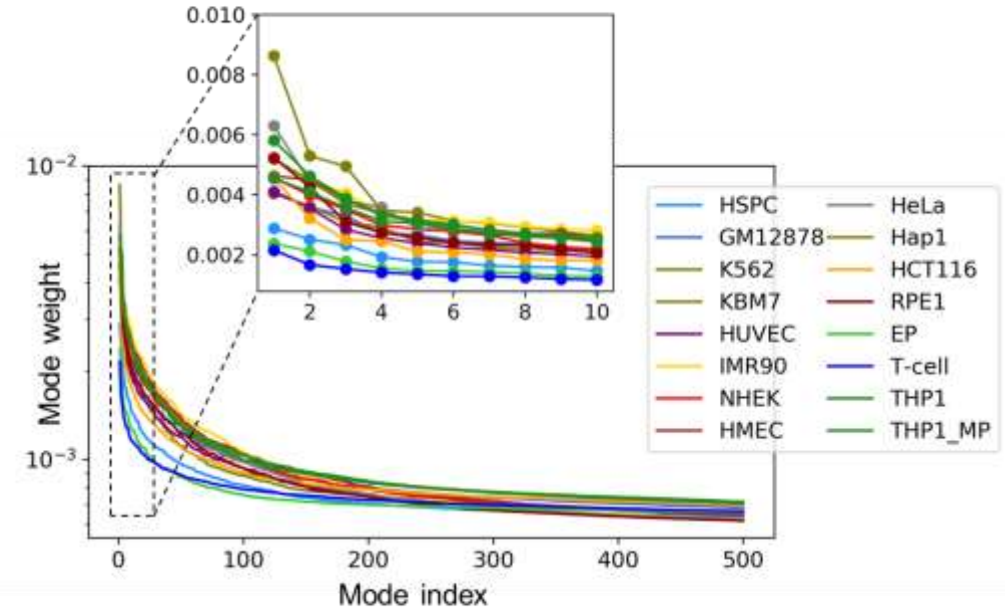

Figure S1. Statistical weights of first 500 modes calculated for chromosome 17 across cell lines. Results are shown on a logarithm scale to emphasize the differences among the cells. *Inset* shows the portion of the curves for the softest 10 modes (on a linear scale along the y-axis). Mode weights are calculated based on Eq. 8.

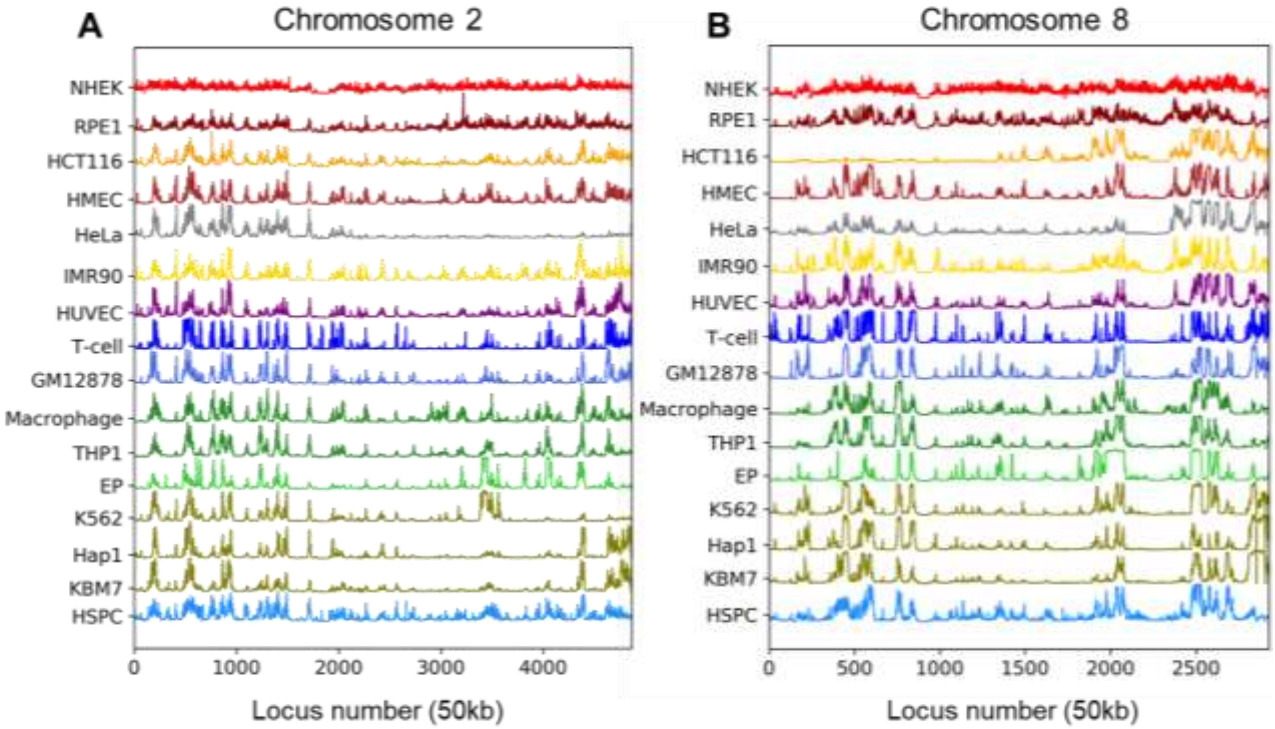

Figure S2. Mobility profiles obtained for different chromosomes and cell lines. **(A)** Mobility profiles of genomic loci computed based on first 500 modes obtained for chromosome 2. **(B)** Same results for chromosome 8.

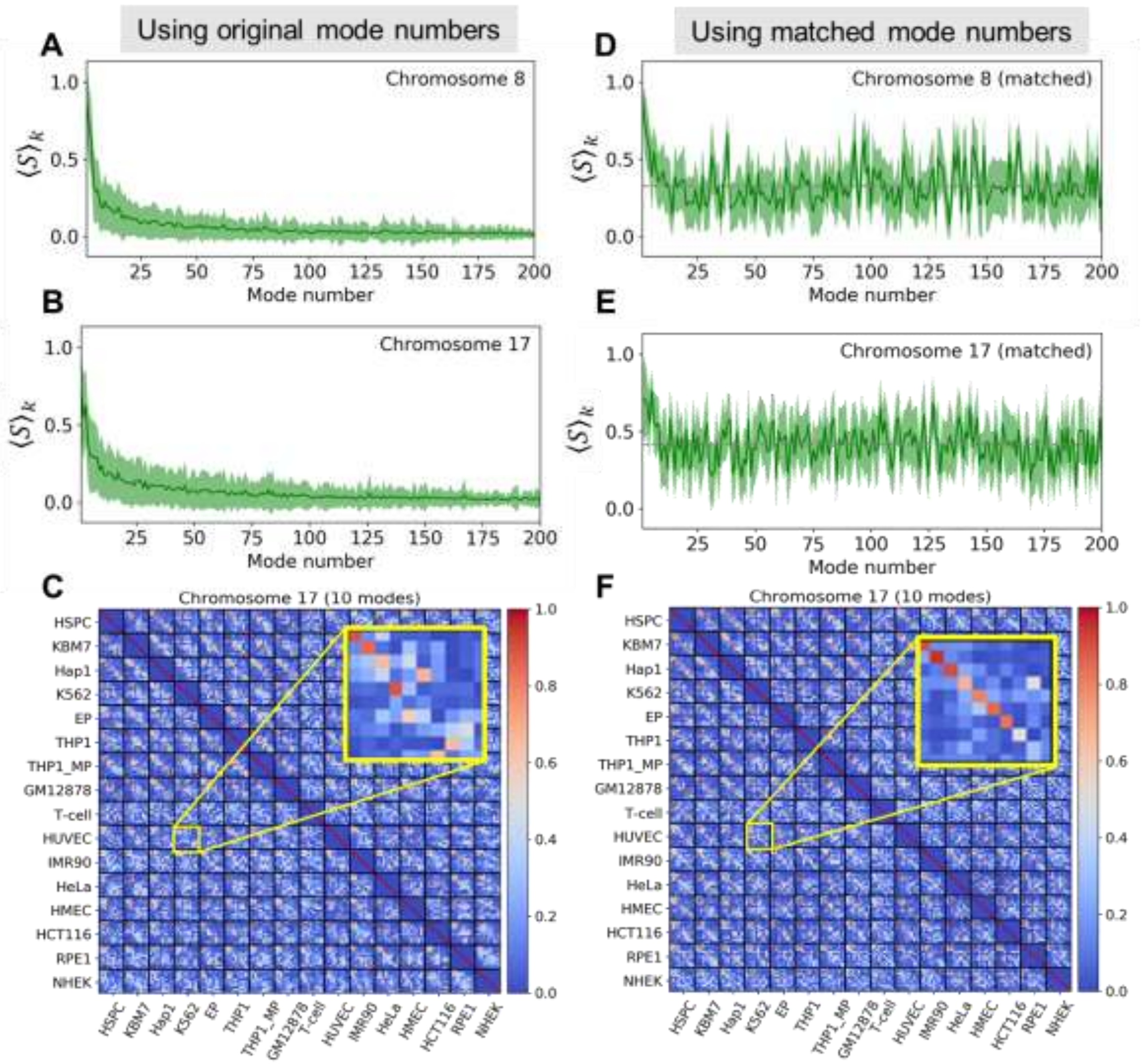

Figure S3. Mode conservation profiles and mode-mode overlaps for chromosomes 8 and 17 before and after matching mode numbers. The results for mode k ($1 \leq k \leq 200$) averaged over all pairs of sixteen cell lines obtained before (**A-C)** and after (**D-F**) matching mode numbers for chromosomes 8 (**A, D**) and 17 (**B-C** and **E-F**) are displayed. The *solid green curve* and the *lighter shade* show the mean conservation profile of the modes and the standard deviations, respectively. Results in panels **A-B** are obtained using the original modes and those in panels **D-E** using the equivalent modes identified as the best matches to the top 500 modes of HSPC selected as reference. The *gray dashed lines in* **D-E** indicate the mean values of the overlaps (0.430 and 0.350, respectively). Higher conservation is achieved by selecting equivalent modes from pre-existing modes the prior probabilities of which differ among different types of cells. Detailed examination of mode-mode overlaps illustrated for the top 10 modes accessible to chromosome 17 (shown for each pair of cells in 10×10 off-diagonal blocks/submatrices) reveal the mismatches between mode numbers (see inset in panel **C** for the enlarged block corresponding to HUVEC-K562 pair), which are eliminated after reordering the modes (inset in panel **F**).

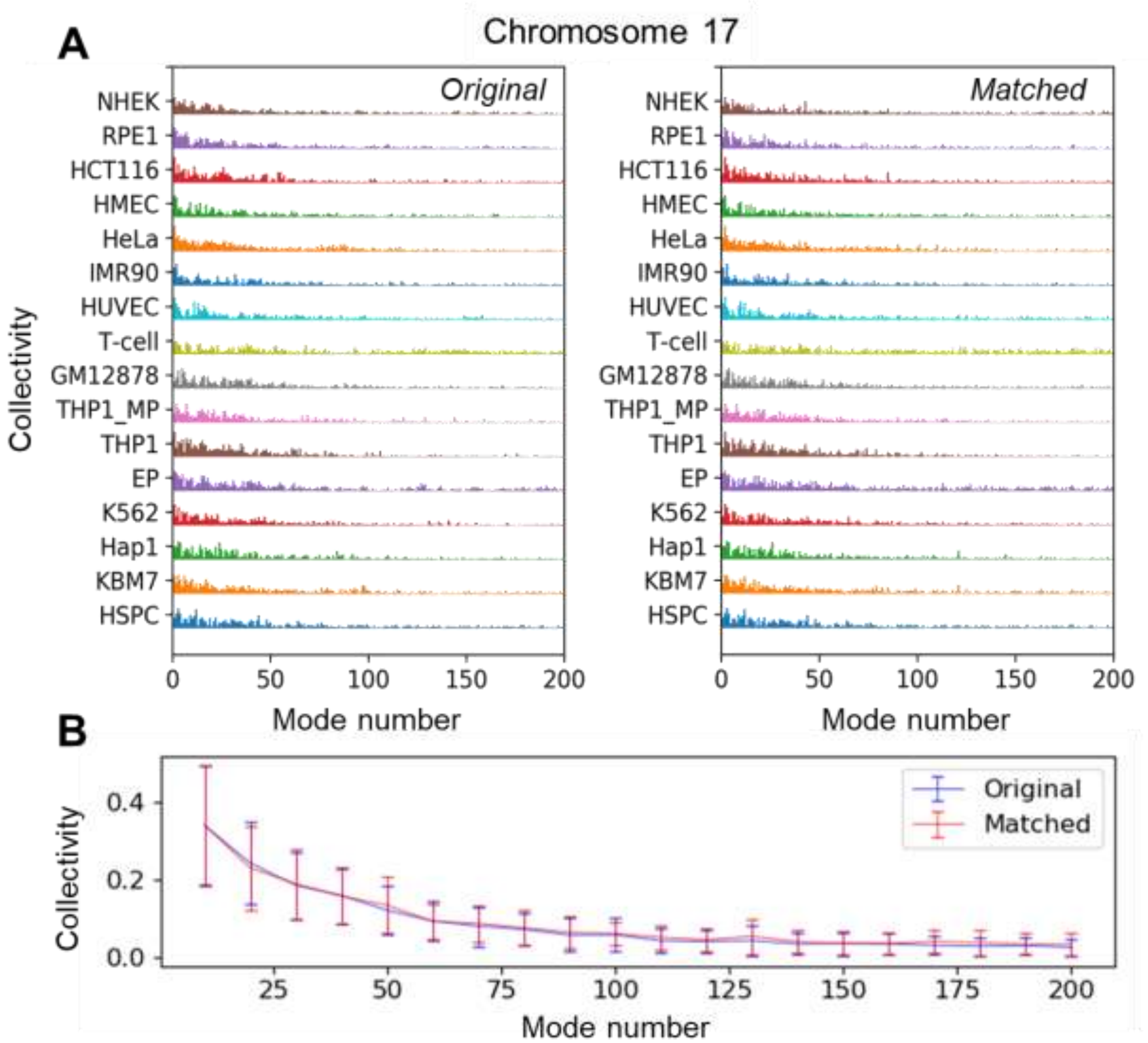

Figure S4. Collectivity profiles of GNM modes illustrated for chromosome 17 loci. **(A)** The bars display the degree of collectivity of each mode, in the range $1 \leq k \leq 200$, obtained for all cell types, before (*left, original*) and after matching the modes to those of the reference, HSPC (*right, matched*). **(B)** Mode collectivities averaged over all cells and plotted based on subsets of 10 modes demonstrate that the two sets display comparable distribution of collectivity in general.

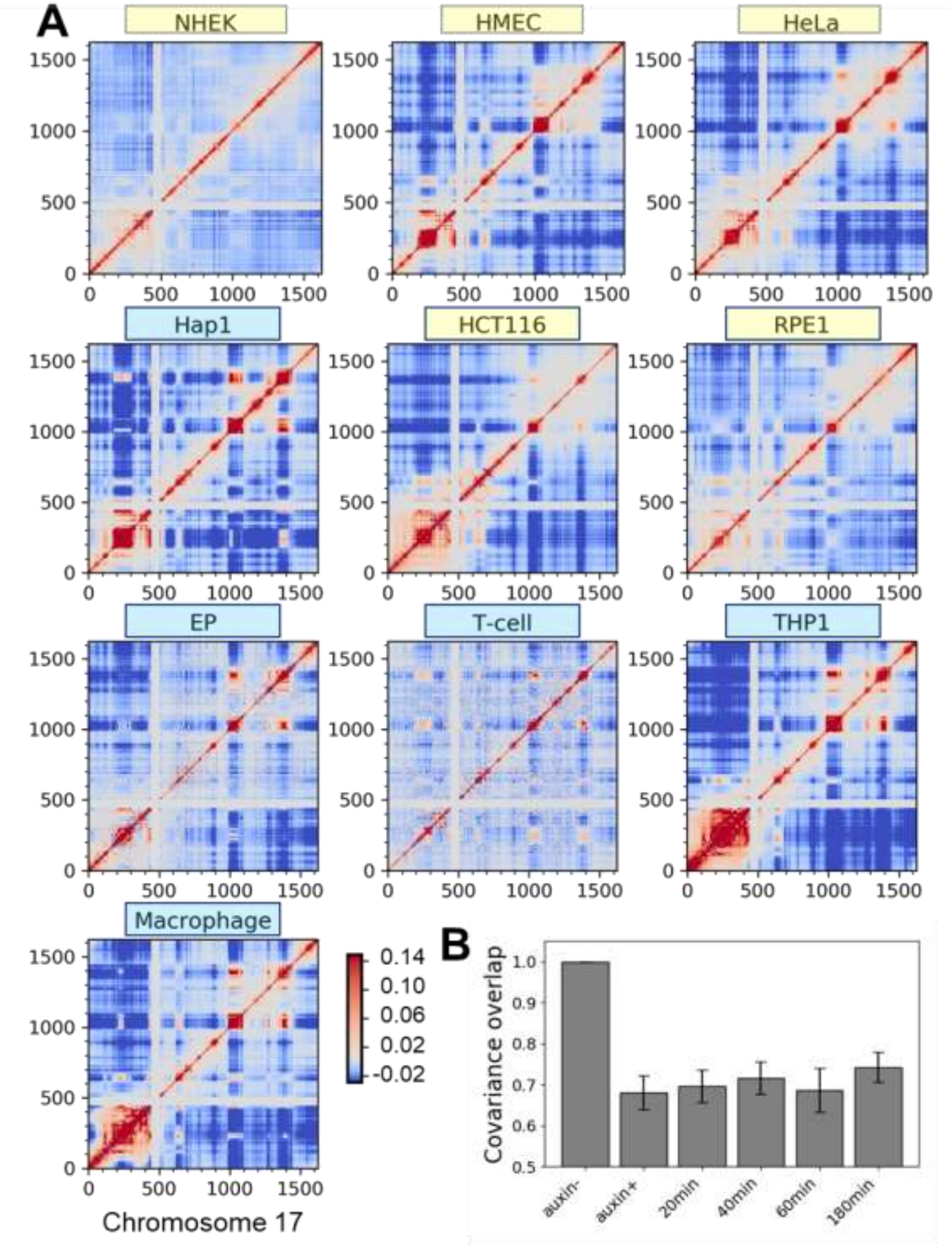

Figure S5. (A) Directional cross-correlation maps for chromosome 17 loci obtained for the indicated ten cell types, complementing those (six cell types) presented in **Figure 5A**. See caption for **Figure 5**. **(B)** Average overlaps between the covariance matrices computed based on Hi-C maps at each time point after the treatment of auxin and those computed based on Hi-C maps for normal HCT116 (1). Standard deviations are shown by *error bars*.

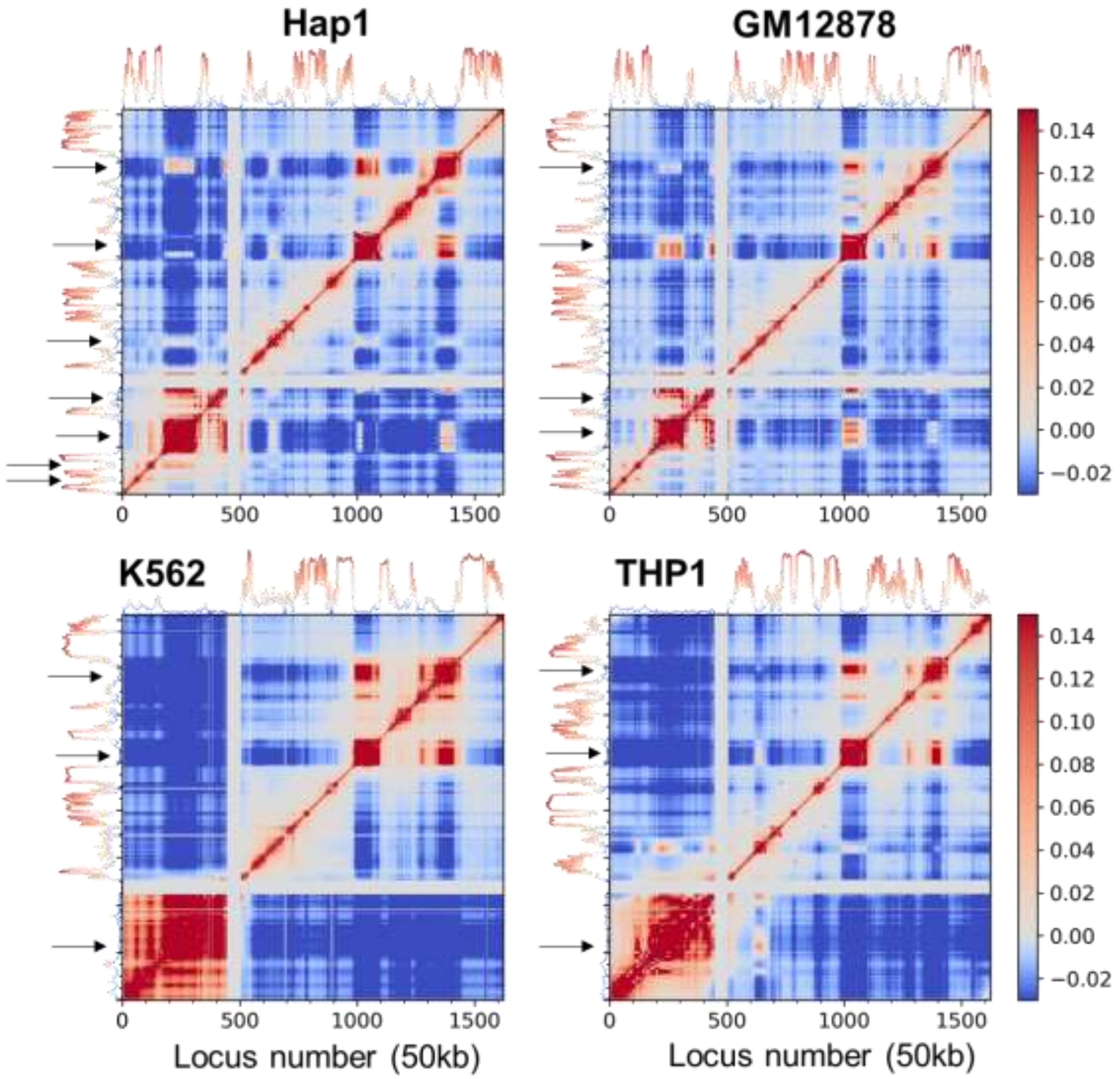

Figure S6. Directional cross-correlation maps aligned with MSFs illustrated for four cell types. The heat maps display the correlation cosines (see Eq. 4) for chromosome 17 of the different types of cells (labelled), based on 500 softest modes. *Black arrows* along the *left ordinate* show examples of regions that exhibit high directional correlations while their mobility is low, indicative of severe spatial restrictions (minima in mobility profiles) constraining the loci to move together, or to be rigidly held together while undergoing small fluctuations. See also **Figures 5** and **S5** for additional information.

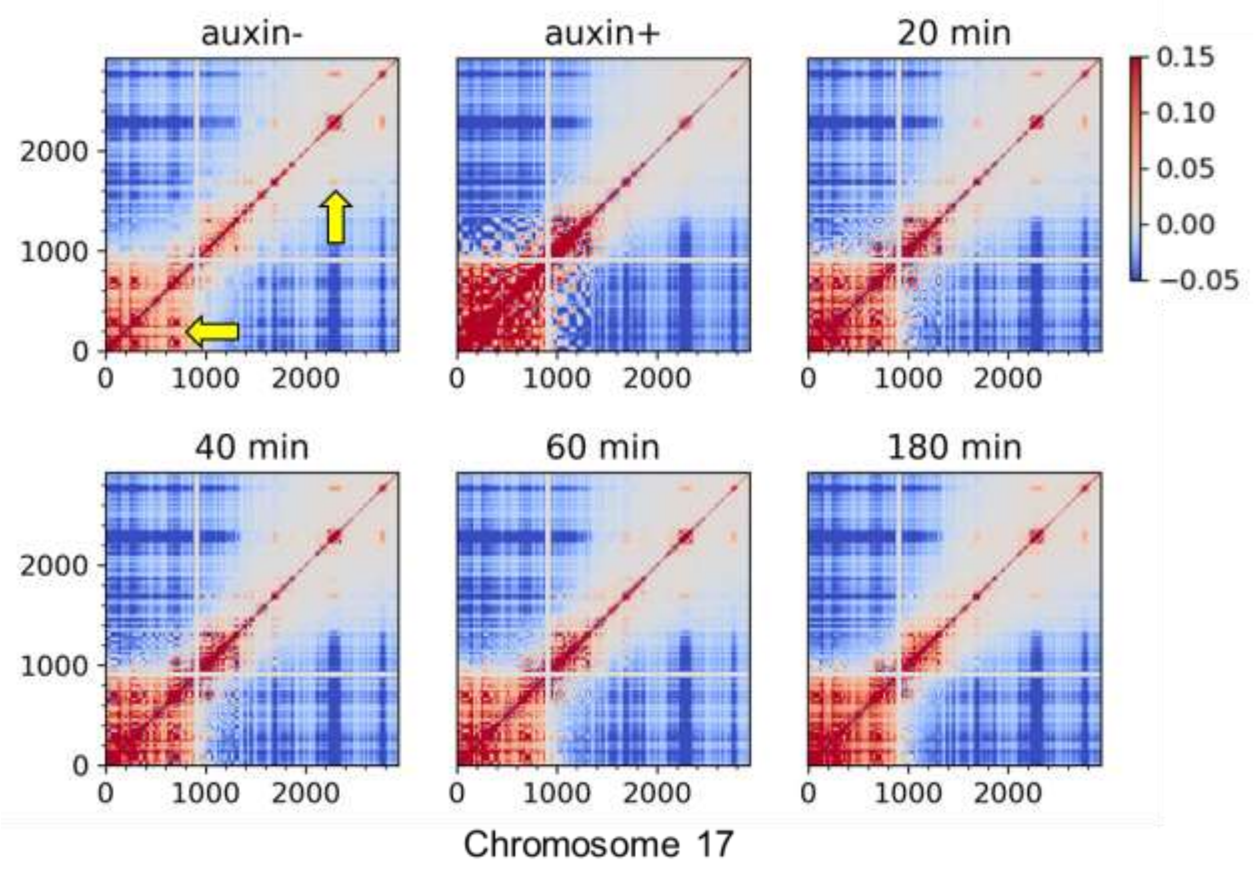

Figure S7. Covariance matrices for chromosome 17 of HCT116 cells before and after auxin treatment, and 20, 40, 60, 180 minutes after auxin has been withdrawn. It can be seen, indicated by *yellow arrows*, loci interactions are greatly weakened or disrupted after auxin treatment (compare the matrix for auxin+ with that for auxin-). These interactions are gradually restored during time after withdrawn (compare the matrices for 20, 40, 60 and 180 minutes). Hi-C data are obtained from (1). See also **Figure S5B**.

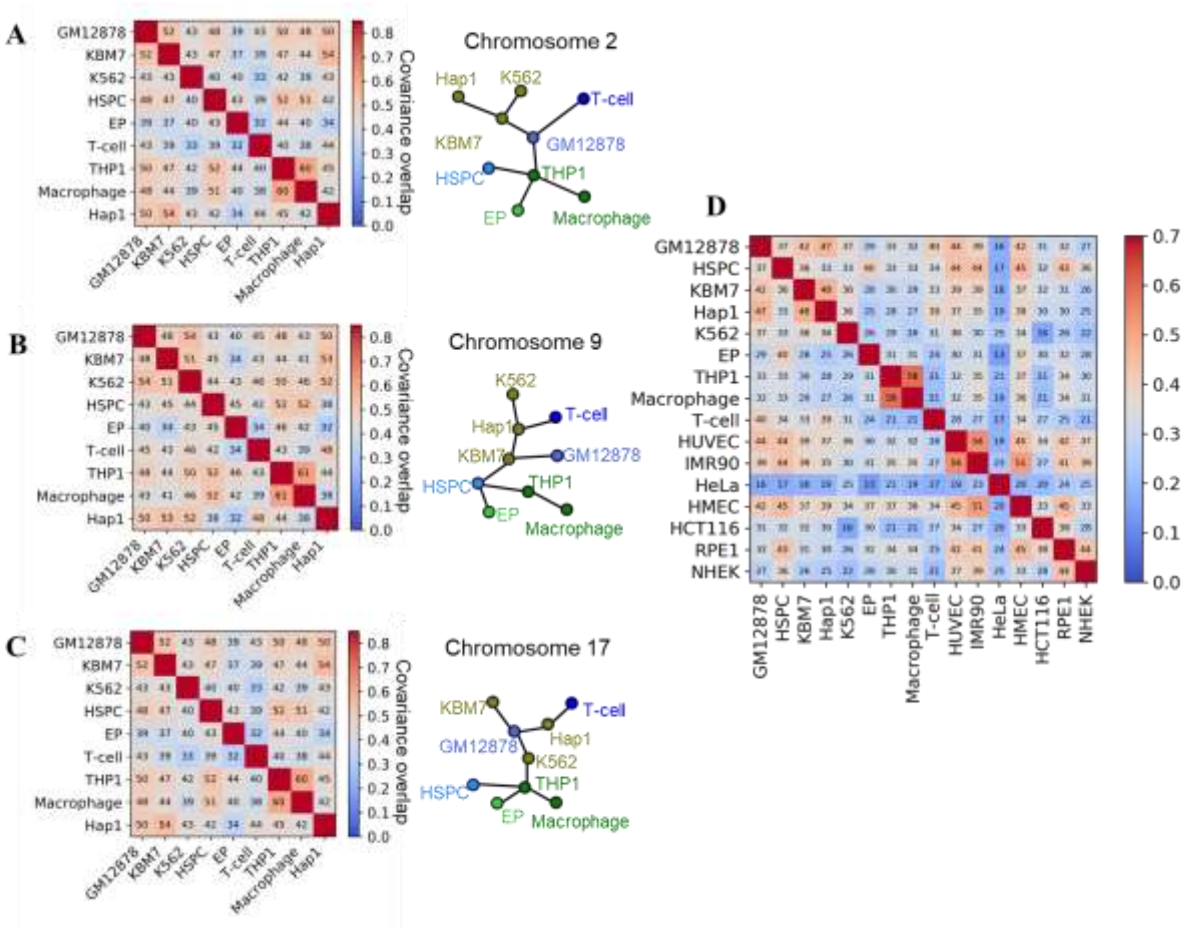

Figure S8. Examples (chromosome 2, 9, 17) showing minimum spanning trees constructed based on the overlaps among covariance matrices obtained for hematopoietic cells. (A) Pairwise covariance overlaps calculated for hematopoietic cell lines (*left*, numbers show the overlap as percentages) and the constructed MST (*right*). Results are obtained for chromosome 17. **(B)** Same results for chromosome 9. **(C)** Same results for chromosome 17. **(D)** Minimum covariance overlaps across all chromosomes obtained for all cell pairs. The numbers show the overlap as percentages.

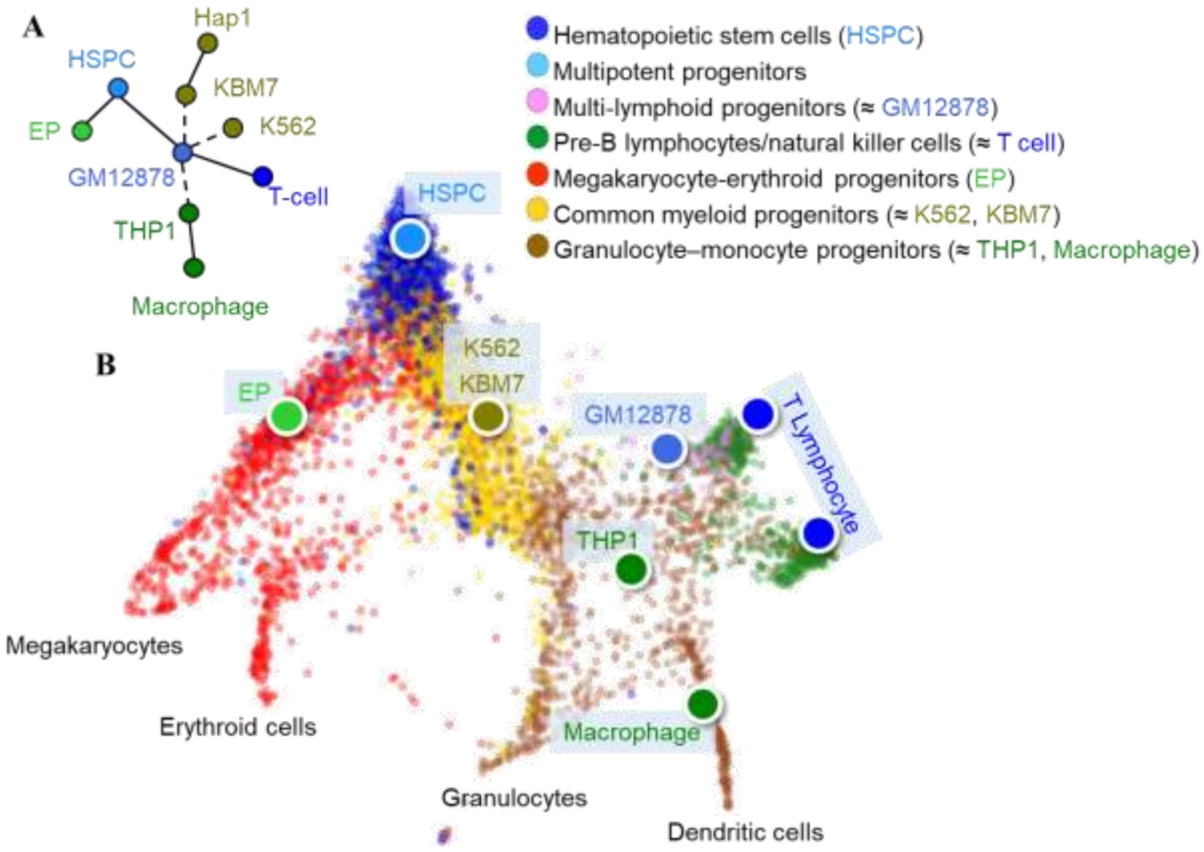

Figure S9. (A) The same tree as in **Figure 5D**, reoriented for the convenience of comparison with panel **B**. **(B)** *k*-nearest neighbor-based clustering/visualization of single-cell RNA-seq data of hemopoietic progenitors, adapted from (2). Cell lines that used in this study are marked by *nodes* at corresponding (approximate) positions on this map and color coded consistently with panel **A**.

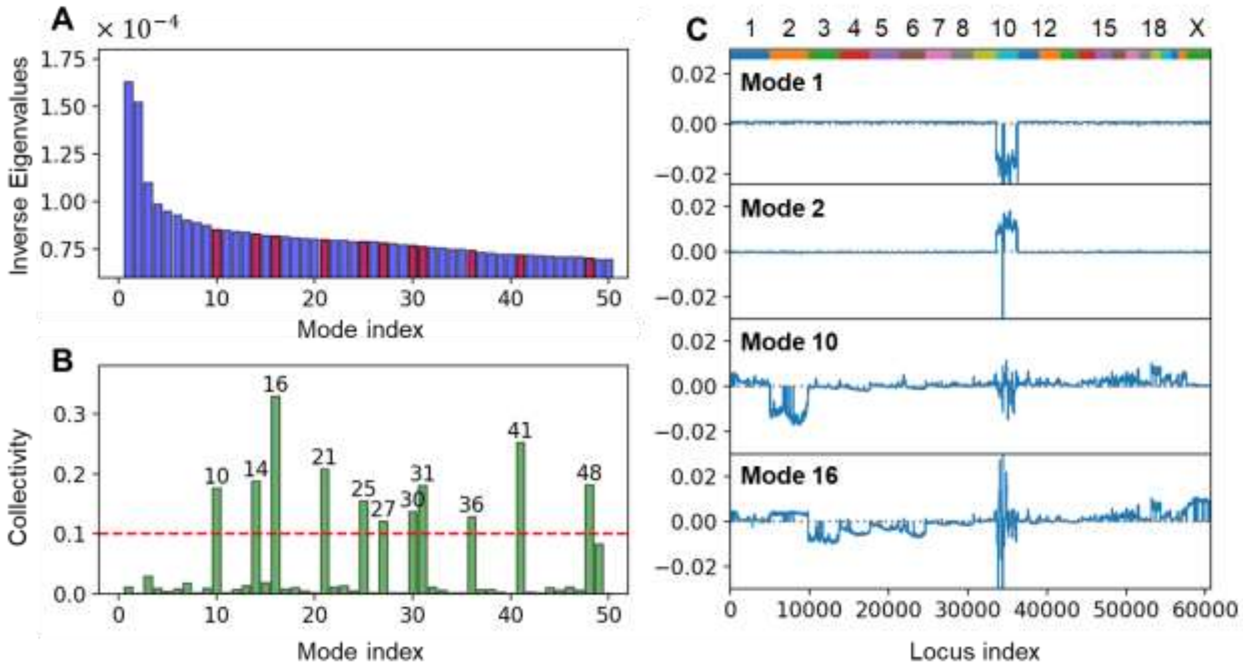

Figure S10. GNM results for GM12878 chromatin reveal the dominance of intrachromosomal motions among soft modes. (A) Statistical weights (inverse eigenvalues) of the softest 50 modes. Note the sharp decrease (frequency gap) after the softest two modes. Modes that have a collectivity higher than 0.1 (see panel **B**) are highlighted by *red bars.* **(B)** Collectivities calculated for the first 50 modes. The *red dashed line* indicates the threshold above which modes display inter-chromosomal couplings. Among the first 100 modes, 14 were found to have a collectivity higher than 0.1 1 (*peaks* in **B**, *red bars* in **A**) and are considered as inter-chromosomal modes. These modes contribute by 14.3% to the total weight of the first 100 modes. The remaining 86 modes were intrachromosomal and contributed by 85.7%. **(C)** The shape of GNM modes 1, 2, 10, and 16. The chromosome ranges are indicated by the colored bars along the upper abscissa. The first two modes (modes 1 and 2) are intra-chromosomal; both involve movements confined to chromosome 10. The latter two (modes 10 and 16) display inter-chromosomal couplings (e.g. between chromosomes 2, 3 and 10 and a few higher chromosomes in mode 10).

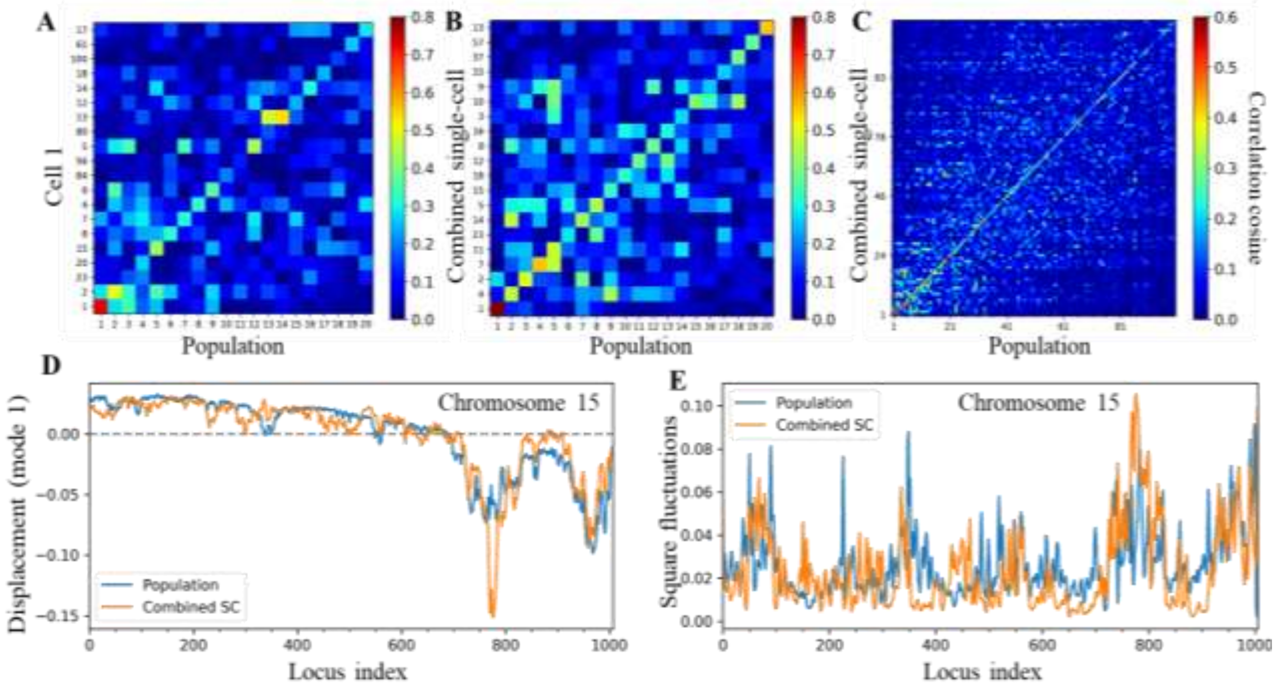

Figure S11. Comparison of chromosomal dynamics obtained by the GNM analysis of single cell Hi-C data and Hi-C data for a cell population of the same type of cells. Hi-C data collected for mouse cells (3) were used as input. GNM results are presented for chromosome 15 in each case. Single cell GNM computations have been performed using the 3D models generated by Stevens et al. (3) **(A)** Cosine similarities between the GNM soft modes calculated for single-cell indexed 1 and those calculated sing the cell population Hi-C data. Modes along the ordinate are matched with respect to the "population". Several modes predicted for the single-cell exhibit similarities to those predicted for the cell population, as can be seen from the entries along the diagonal. (**B**) Same as (**A**), but single-cell data is replaced by 'combined single-cell', after consolidating the Hi-C data from eight individual cells of the same type. (**C**) Same as (**B**), presented for 100 soft modes. **(D)** Shape (eigenvector) of mode 1 obtained from and compared between population and combined single-cell Hi-C. **(E)** Mean-square fluctuations of gene loci on chromosome 15, for the same two systems (see label) calculated using the softest 100 GNM modes. For visual comparison, MSFs were smoothed by running averages over a window of three loci.

Supplementary references

1. Rao, S.S.P., Huang, S.C., Glenn St Hilaire, B., Engreitz, J.M., Perez, E.M., Kieffer-Kwon, K.R., Sanborn, A.L., Johnstone, S.E., Bascom, G.D., Bochkov, I.D. *et al.* (2017) Cohesin Loss Eliminates All Loop Domains. *Cell*, **171**, 305-320 e324.
2. Pellin, D., Loperfido, M., Baricordi, C., Wolock, S.L., Montepeloso, A., Weinberg, O.K., Biffi, A., Klein, A.M. and Biasco, L. (2019) A comprehensive single cell transcriptional landscape of human hematopoietic progenitors. *Nat. Commun.*, **10**, 2395.
3. Stevens, T.J., Lando, D., Basu, S., Atkinson, L.P., Cao, Y., Lee, S.F., Leeb, M., Wohlfahrt, K.J., Boucher, W., O'Shaughnessy-Kirwan, A. *et al.* (2017) 3D structures of individual mammalian genomes studied by single-cell Hi-C. *Nature*, **544**, 59-64.
